# From Methane to Methanol: Pd-iC-CeO_2_ Catalysts Engineered for High Selectivity via Mechanochemical Synthesis

**DOI:** 10.1021/jacs.4c04815

**Published:** 2024-08-15

**Authors:** Juan D. Jiménez, Pablo G. Lustemberg, Maila Danielis, Estefanía Fernández-Villanueva, Sooyeon Hwang, Iradwikanari Waluyo, Adrian Hunt, Dominik Wierzbicki, Jie Zhang, Long Qi, Alessandro Trovarelli, José A. Rodriguez, Sara Colussi, M. Verónica Ganduglia-Pirovano, Sanjaya D. Senanayake

**Affiliations:** † Chemistry Division, Brookhaven National Laboratory, Upton, New York 11973, United States; ‡ CSIC, Instituto de Catálisis y Petroleoquímica, C/Marie Curie 2, 28049 Madrid, Spain; § Polytechnic Department, University of Udine and INSTM, Via del Cotonificio 108, 33100 Udine, Italy; ∥ Universitat Politècnica de València, Camí de Vera s/n, 46022 València, Spain; ⊥ Center for Functional Nanomaterials, Brookhaven National Laboratory, Upton, New York 11973, United States; # National Synchrotron Light Source II, Brookhaven National Laboratory, Upton, New York 11973, United States; ∇ Ames National Laboratory, Iowa State University, Ames, Iowa 50011, United States; ○ Department of Chemistry, State University of New York Stony Brook, Stony Brook, New York 11794, United States

## Abstract

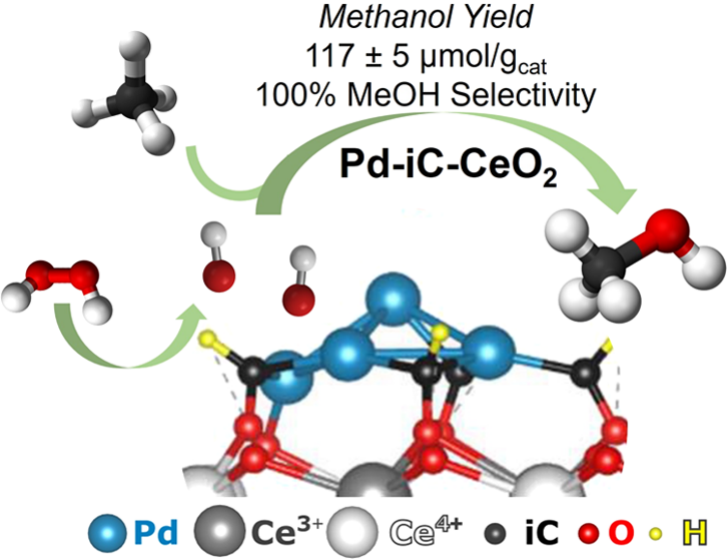

In the pursuit of selective conversion of methane directly to methanol in the liquid-phase, a common challenge is the concurrent formation of undesirable liquid oxygenates or combustion byproducts. However, we demonstrate that monometallic Pd-CeO_2_ catalysts, modified by carbon, created by a simple mechanochemical synthesis method exhibit 100% selectivity toward methanol at 75 °C, using hydrogen peroxide as oxidizing agent. The solvent free synthesis yields a distinctive Pd-iC-CeO_2_ interface, where interfacial carbon (iC) modulates metal-oxide interactions and facilitates tandem methane activation and peroxide decomposition, thus resulting in an exclusive methanol selectivity of 100% with a yield of 117 μmol/g_cat_ at 75 °C. Notably, solvent interactions of H_2_O_2_ (aq) were found to be critical for methanol selectivity through a density functional theory (DFT)-simulated Eley–Rideal-like mechanism. This mechanism uniquely enables the direct conversion of methane into methanol via a solid–liquid–gas process.

## Introduction

The direct utilization of natural gas, which is primarily composed of methane (CH_4_) and is a potent greenhouse gas, presents a significant scientific challenge.^1^ This challenge is mainly attributed to the strong C–H bonds of methane (104 kcal/mol), which tend to drive methanol to overoxidation into undesirable CO_2_ rather than useful chemicals. The direct conversion of natural gas into liquid fuels, such as methanol (CH_3_OH), through a single-step process, is an attractive option. This method offers several advantages, including the ease of transporting liquid oxygenates compared to pressurized or liquified gases from highly localized and remote natural gas well locations. Biomimetic approaches that mimic nature’s own methane monooxygenase enzymes, typically employing zeolites, often face limitations such as high temperature requirements, which lead to selectivity toward undesirable combustion products.^2^ In contrast, heterogeneous catalysts based on metal oxides have shown promise in directing selectivity toward methanol using various active materials like Rh/ZrO_2,_^3^ Pd/TiO_2,_^4^ Ni/CeO_2,_^5^ CeO_2_/CuO/Cu(111),^6^ Ir-based systems^7,8^ and FeO_*x*_/TiO_2._^9^ They offer advantages like a high dispersion of the active metal sites and facilitate intimate contact between the reducible support and metal sites.^10^

Gas phase conversion of methane to methanol generally consist of sequentially cycled O_2_ activation, CH_4_ reaction, and a final H_2_O purge step,^11^ each at distinctly different operating conditions, while liquid phase methane to methanol is facilitated by being a one pot synthesis at a fixed condition in either H_2_O_2_ (aq)^12^ or an O_2_/H_2_O environment.^8^ Studies aimed at elucidating the reaction mechanism of direct methane to methanol (MtM) conversion in the presence of gas-phase H_2_O_2_ or O_2_ using metal/oxide catalysts have made significant progress.^3^ These studies have identified AuPd active sites capable of generating peroxide in situ,^13,14^ explore the influence of oxidants and CO on reaction intermediates, and revealed distinct mechanisms when using H_2_O_2_ as an oxidant^8,15^ versus molecular oxygen.^16^ With peroxide, it is generally accepted that two independent catalytic cycles occur. The first involves the decomposition of hydrogen peroxide, resulting in the formation of 2OH* species. Simultaneously, methane is converted into methyl, CH_3_, which undergoes sequential oxidation to methanol and eventually CO_2_ as the final product at higher temperatures and longer reaction times.^17^ A similar stepwise conversion could take place in the liquid phase for the MtM process on Cu_1_–Ag_1_/ZSM-5 catalysts.^18,19^ However, in the case of liquid phase batch mode reactions, the precise mechanism of methane to methanol remains elusive due to the challenge of characterizing the catalyst and reaction intermediates in the liquid phase. Furthermore, the possible existence of a metal–carbon-oxide interface could affect the selectivity for methanol production, as seen over Au/HZSM-5 with carbon additives.^20^

In this study, we have developed Pd based catalysts for the MtM reaction in a liquid-phase environment, particularly in the presence of H_2_O_2_ and H_2_O. These catalysts are synthesized using a straightforward mechanochemical method,^21−23^ enhancing the formation of a stable and active interfacial carbon (iC) layer. This iC layer creates a distinctive Pd-iC-CeO_2_ interface, a pivotal element in preventing the overactivation of hydrogen peroxide and modulating the overall oxidative potential of the catalyst to promote liquid oxygenate products, chiefly methanol. Our research employs in situ attenuated total reflectance infrared spectroscopy (ATR-IR), X-ray photoelectron spectroscopy (XPS), near-edge X-ray absorption fine structure spectroscopy (NEXAFS), and X-ray absorption spectroscopy (XAFS). Additionally, we integrate a comprehensive understanding of the reaction profile through density functional theory (DFT) calculations. The primary focus of our study centers on exploring the impact of the metal–carbon-oxide interface in mechanochemically prepared Pd acetate-CeO_2_ catalysts, denoted as Pd-iC-CeO_2_.

## Results and Discussion

### Catalytic Performance of Pd-iC-CeO_2_: Balancing Methane Oxidation and H_2_O_2_ Decomposition

**Figure 1:**
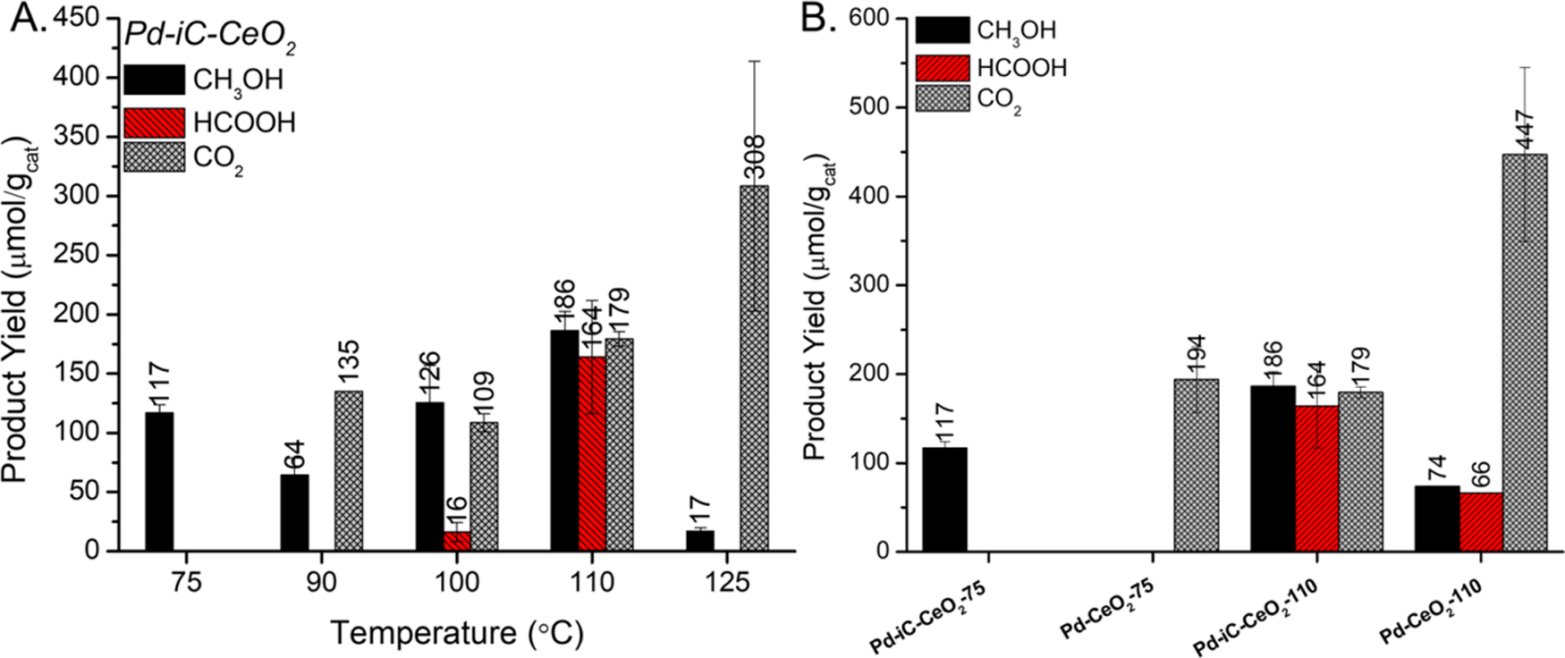
Reactivity of Pd-CeO_2_ materials for direct methane to methanol conversion in a batch mode reactor. (A) Methanol, HCOOH and CO_2_ yield as a function of temperature (75–125 °C) for Pd-iC-CeO_2_, (B) comparison between reference materials at 75 or 110 °C. Reaction conditions: 25 mg of catalyst, 1 h reaction time, 15 mL of 0.5 M H_2_O_2_ (aq), 20 bar initial pressure of 20% CH_4_ in Ar balance, 800 rpm mixing.

Pd-iC-CeO_2_ shows a 100% selectivity toward methanol at 75 °C (Figure 1A), with no detectable formation of additional oxygenate species. Upon heating to higher temperatures, the reaction favors the production of formic acid, HCOOH, and carbon dioxide, CO_2_, without the observation of complex oxygenates such as CH_3_OOH and dimethyl ether for the milled catalyst (Table S1). The general trend in reactivity as a function of temperature shows that, as the temperature increases, there is a sequential increase in higher order oxygenates following the trend of CH_4_ > CH_3_OH > HCOOH > CO_2_ under the given reaction conditions with a corresponding increase in the overall rate of methane reaction, indicated by the cumulative increase in total products. The novelty inherent in the Pd-iC-CeO_2_ catalyst lies in the formation of a unique Pd-iC-Ce interface, which plays a crucial role in dampening the hydrogen peroxide decomposition rates and subsequent formation of higher-order oxygenates. In the absence of the iC layer, Pd-CeO_2_ shows no selectivity toward methanol at 75 °C and minimal liquid oxygenates at 110 °C (Figure 1B).

**Figure 2:**
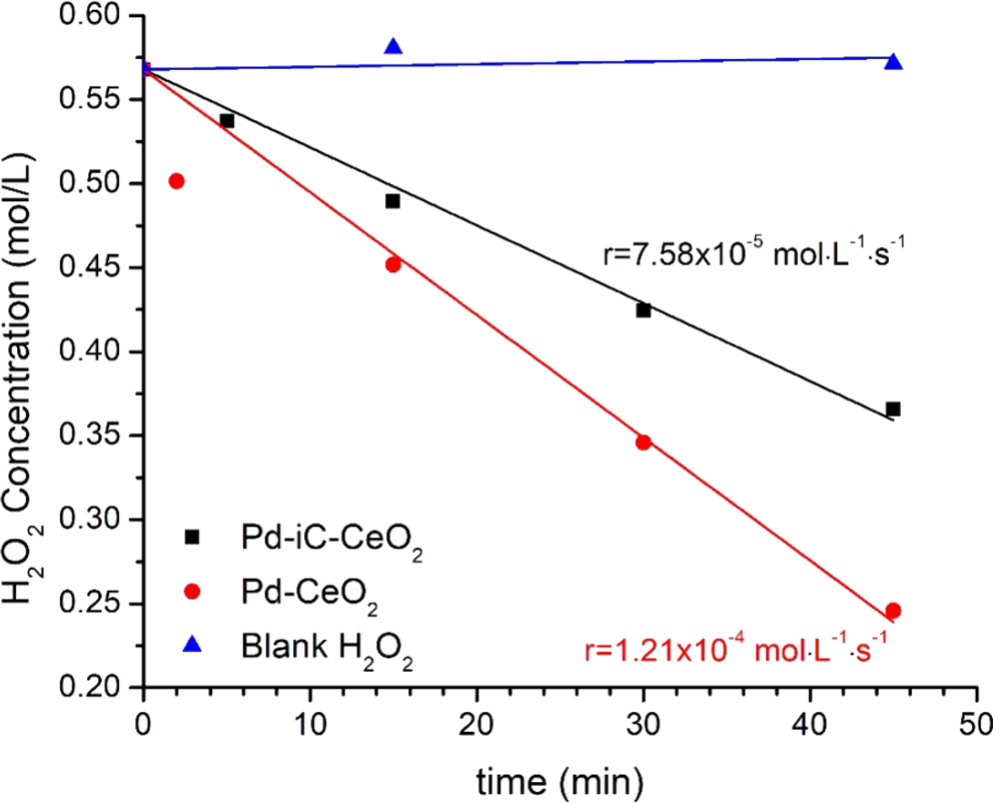
Experimental H_2_O_2_ decomposition rates evaluated at ambient pressure and temperature over Pd-iC-CeO_2_, Pd-CeO_2_, and blank H_2_O_2_ (absence of catalysts) in air. H_2_O_2_ concentrations are determined via acidified cerium(IV) sulfate titration.

Notably, the Pd-iC-CeO_2_ catalyst exhibits a hydrogen peroxide decomposition rate that is a factor of 1.5 lower than that of the conventional Pd-CeO_2_ catalyst (Figure 2), with the rate of peroxide decomposition of 7.58 × 10^–5^ vs 1.21 × 10^–4^ mol/L/s on Pd-iC-CeO_2_ and Pd-CeO_2_, respectively. The carbon present in the Pd-iC-CeO_2_ interface blocks sites involved in the decomposition of H_2_O_2_, consistent with the theoretical results that will be presented later, which revealed not only a factor of 2.5 stronger H_2_O_2_ binding on Pd-CeO_2_ compared to Pd-iC-CeO_2_ (−1.27 vs −0.50 eV for Pd-CeO_2_ and Pd-iC-CeO_2_, respectively) but also a factor of 1.14 times stronger exothermicity in the H_2_O_2_ to 2OH reaction. Consequently, this leads to a reduced availability of oxidant for the decomposition of methanol. The ability to independently tune the H_2_O_2_ decomposition rate and the CH_4_ activation cycle is critical to achieve ideal selectivity. Through comparative analysis at 75 °C with reference catalysts (Figure S1) the need for the precise moiety of Pd-iC-CeO_2_ was expanded by deconvoluting the contributions from each individual component and combination thereof. In this comparison Pd(OAc)_2_ highlights the possibility of a palladium acetate species driving the chemistry, which yielded only methy peroxyl. Pd-iC-SiO_2_, obtained via the mechanochemical synthesis of Pd-iC with SiO_2_ as the support instead of CeO_2_, where SiO_2_ is taken to be an inert support, was used to investigate the Pd-iC interface activity without CeO_2_, which ultimately favored overoxidation into CO_2_. Finally, Pd-CeO_2_ assessed the performance of the pure Pd-CeO_2_ interface in the absence of the iC interlayer, which led to the lowest selectivity toward oxygenates and purely CO_2_. This underscores the necessity of Pd, carbon, and CeO_2_ synergy at the Pd-iC-CeO_2_ interface for selective methanol formation, where the protection of methanol is often stated as a critical process parameter,^24^ here we present the possibility of dampening the oxidative potential of the site itself via the incorporation of interfacial carbon (iC) to promote selective conversion toward methanol. This results in a novel catalyst interface that yields competitive rates of reaction with other state-of-the-art methane to methanol catalysts, shown in Table S2.^3,4,8,14,25^

**Figure 3:**
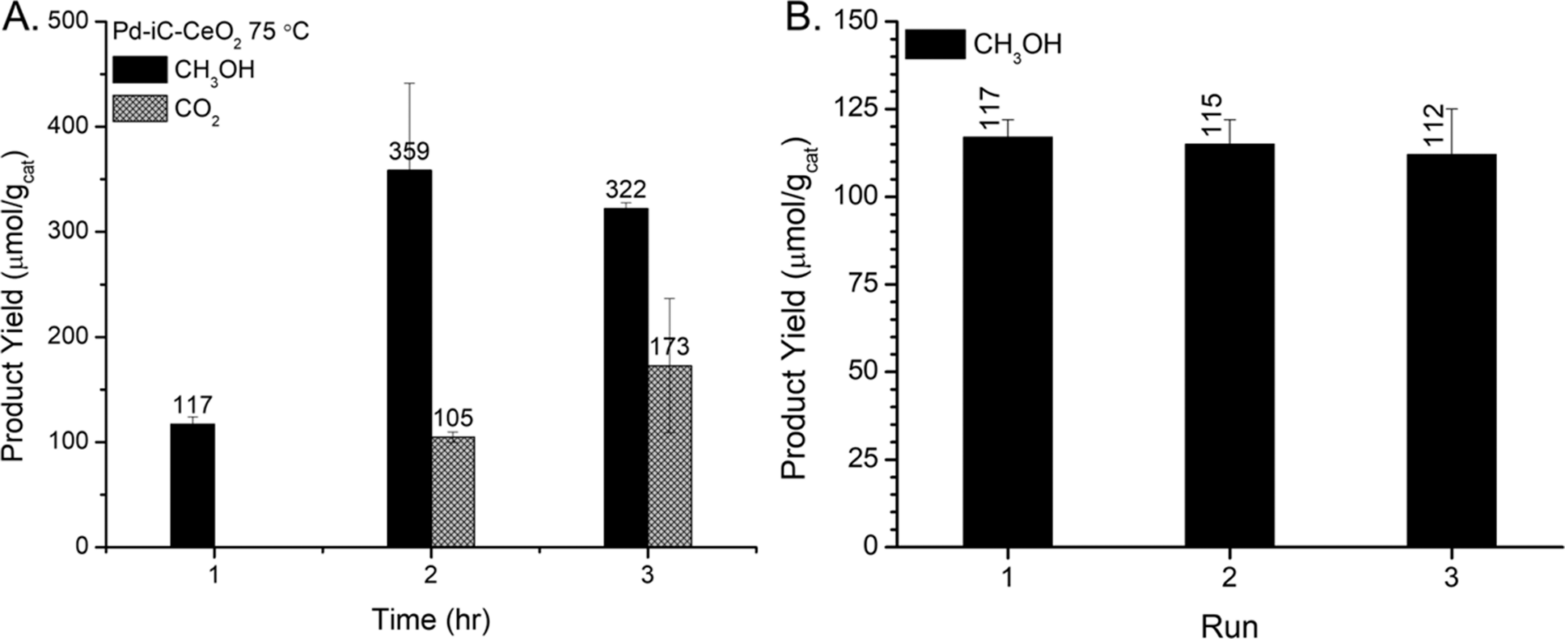
Direct methane to methanol stability testing over Pd-iC-CeO_2_ (A) time dependent product yield at 75 °C for Pd-iC-CeO_2_ from 1–3 h, (B) recyclability tests at 75 °C for Pd-iC-CeO_2_. Reaction conditions: 25 mg of catalyst, 15 mL of 0.5 M H_2_O_2_ (aq), 20 bar initial pressure of 20% CH_4_ in Ar balance, 800 rpm mixing.

**Figure 4:**
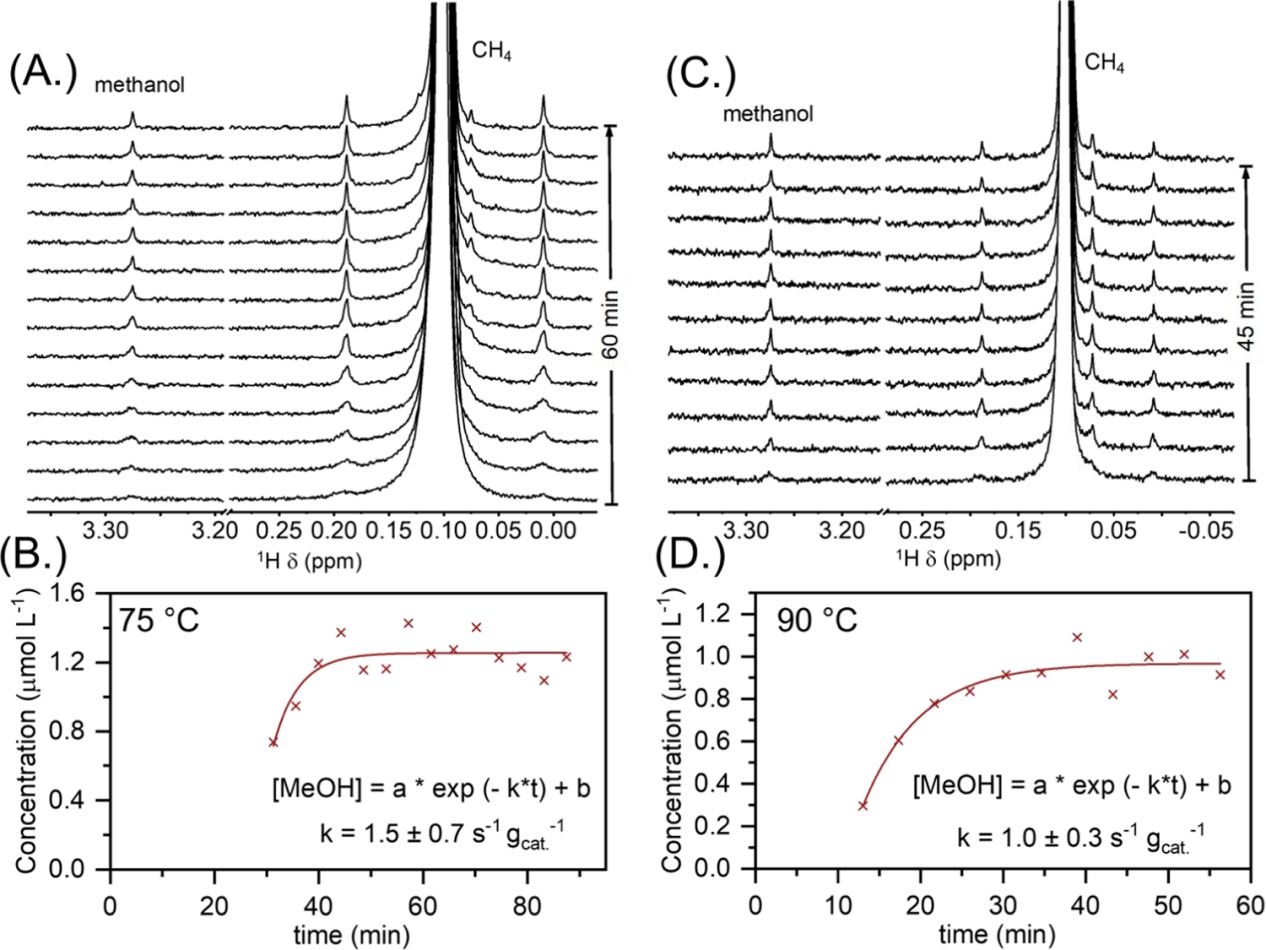
In situ solution NMR studies of direct methane to methanol reaction over Pd-iC-CeO_2_. Arrays of the ^1^H NMR spectra (4 min 13 s per spectrum) at (A) 75 and (C) 90 °C. Kinetic analysis of time-resolved NMR spectra at (B) 75 and (D) 90 °C. The first-order rate law was assumed to curvefit the time-resolved concentration profile to obtain the rate constant. Reaction conditions: Pd-iC-CeO_2_ catalyst (2.3 mg), 0.5 M H_2_O_2_ in H_2_O/D_2_O solution (3% H_2_O; 97% D_2_O; 400 μL), 5 bar methane, 75 or 90 °C.

Moreover, the product distribution as a function of time (Figure 3A) exhibits the expected increase in methanol yield. However, with prolonged reaction time, selectivity diminishes, attributed to the formation of CO_2_, which is likely due to either exposure of distinct active sites or sequential overoxidation. The plateaued methanol production rate was corroborated via in situ ^1^H NMR which monitored the methanol production rate in a methane pressurized sealed NMR vessel, modeling the batch reactor configuration at 75 and 90 °C (Figure 4A,C), which showed that after 1 h under reaction conditions at 75 °C the methanol reaches a maximum (Figure 4A). The first-order fitted rate constant for methanol production via in situ NMR was found to be 1.5 ± 0.7 and 1.0 ± 0.3 s^–1^ g_cat_^–1^ for 75 and 90 °C, respectively (Figure 4B,D), where the induction period before data collection represents the system coming up to temperature. The decrease in the methanol rate constant is consistent with the kinetics which show that the rate of methanol production decreases at 90 °C, in favor of CO_2_ production, where the rate constants are derived from in situ NMR are based on methanol production rate, not total CH_4_ rate of reaction. This highlights that the methanol does not appreciably decrease as a function of time, indicating it is not oxidized into higher order oxygenates and/or combustion products sequentially but rather combustion occurs rapidly over distinct active sites that differ from the sites required for methanol formation. This further highlights the importance of not just protecting the methanol intermediates^24^ but the active site itself to preserve the favorable surface motifs. Notably, the beneficial properties of the mechanochemically prepared Pd-iC-CeO_2_ catalyst persist through multiple catalytic cycles (Figure 3B), where the activity remains within error of 117 ± 10 μmol/g_cat_. The catalyst consistently maintains 100% methanol selectivity even after three consecutive reaction cycles, highlighting the absence of significant Pd leaching. This absence is crucial, as Pd leaching could otherwise lead to a substantial loss in reactivity. Furthermore, the catalyst’s dispersion remains largely unchanged, starting at 22% and decreasing to 17% after 3 h reaction time, suggesting that the change in reactivity results from changes in the nature of the active site, rather than merely the number of sites.

### Direct Influence of iC Layer on Pd-iC-CeO_2_ for Methane to Methanol Reactivity

**Figure 5:**
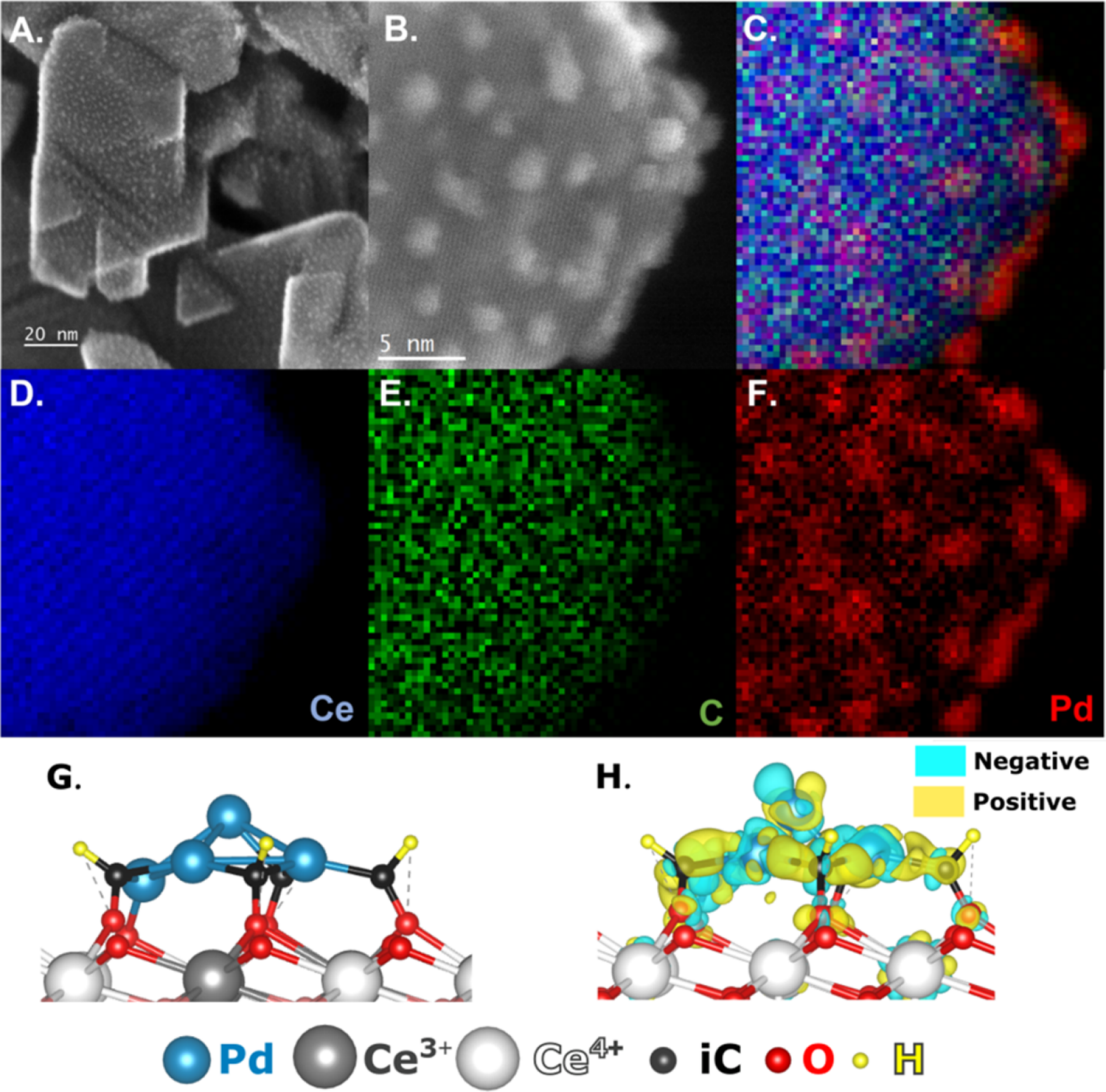
Secondary electron (SE) STEM imaging of Pd-iC-CeO_2_ for (A) after 3 consecutive reaction cycles (B) STEM-EELS inset (C) STEM-EELS combined elemental mapping, (D–F) Ce, C, and Pd elemental mapping, respectively. Reaction conditions: 25 mg of catalyst, 75 °C for 1 h, 15 mL of 0.5 M H_2_O_2_ (aq), 20 bar initial pressure of 20%CH_4_ in Ar balance, 800 rpm mixing. Theoretical model of Pd-iC-CeO_2_ showing the (G) side view and (H) charge density difference plot, positive/negative differences are visually represented with yellow/cyan, respectively. Color coding: Pd atoms are light blue, Ce^4+^ white, Ce^3+^ gray, O atoms of the first layer red, while those of the second layer are light red, iC atoms black and H atoms yellow.

The distinctive Pd-iC-CeO_2_ interface (Figure 5A–F) is highly stable, shown by the intimate contact between the Pd, Ce, and iC species being preserved after three consecutive reaction cycles. The migration of the iC layers toward the Pd is dependent on the reaction time, where after the optimized 1 h reaction time the catalyst structure is largely preserved (Figure S2), but after a 3 h reaction time the carbon is notably seen to migrate specifically toward the Pd, encompassing the metal and segregating from the CeO_2_ (Figure S3). This segregation of the iC layer toward Pd, resulting in the absence of Pd-iC-CeO_2_ interface is consistent with the observed CO_2_ formation in control experiments where the Pd-iC layer and Pd-CeO_2_ were probed in isolation (see Figure S2), promoting increased CO_2_ formation. This reflects the loss of the Pd-iC-CeO_2_ moiety, favoring the formation of surface Pd-iC, where the Pd-CeO_2_ interface is diminished due to carbon migration onto the Pd. Additionally, the exfoliation of the Pd particles is observed over time, with a decrease in wetting of Pd onto the CeO_2_ support, accompanied by a 30% increase in particle size from 1.8 to 2.4 nm (Figure S4). The palladium particles remain in an amorphous state, confirmed by powder X-ray diffraction (pXRD), which shows no Pd diffraction (Figure S5). Ostwald ripening, evident from the broadened Pd size distribution, is identified as the mechanism for particle growth, possibly resulting in the trapping of Pd adatoms on the CeO_2._^26^

To discern the influence of the iC layer in Pd-iC-CeO_2_, the iC layer was systematically removed via an oxidative pretreatment at 500 °C under air (Figure S6), resulting in a decomposed Pd-CeO_2_ interface that is easily destabilized after reaction (Figures S6 and S7). In line with our evidence that the Pd-iC-CeO_2_ dampens H_2_O_2_ decomposition, the removal of the iC layer via oxidative treatment led to the significant evolution of H_2_O_2_ into O_2_ (g), even at room temperature. Consequently, this inhibited methanol formation at 75 °C due to the absence of available reactive oxidants. After this treatment, Pd-iC-CeO_2_ displays no activity for methane partial oxidation to either CO_2_ or oxygenates, as the C interlayer is stripped, evidenced by thermogravimetric analysis (TGA) analysis (Figure S8). To rule out the possibility of the iC layer independently reacting with H_2_O_2_ to yield oxygenates, which have been reported to participate in the MtM reaction to form oxygenates in carbon containing materials,^20^ the reaction was conducted under identical reaction conditions but pressurized with Argon instead of CH_4_, making iC the only potential source of carbon, which resulted in no oxygenate or CO_2_ formation. This finding indicates that the interfacial carbon was not being consumed to form product, highlighting the importance of synchronizing both the catalytic pathways of H_2_O_2_ decomposition and methane activation while tailoring the active site to favor selective methanol production. In an effort to enhance the selective conversion of methane to methanol and synchronize H_2_O_2_ evolution_,_ secondary metals are often employed alongside Pd, such as AuPd,^14,27^ or MPd (M = Cu, Ni, Mn, Au),^28^ resulting in increased reactivity toward oxygenates.

### Surface Intermediates of Pd-iC-CeO_2_ during Realistic Solvated Methane to Methanol

**Figure 6:**
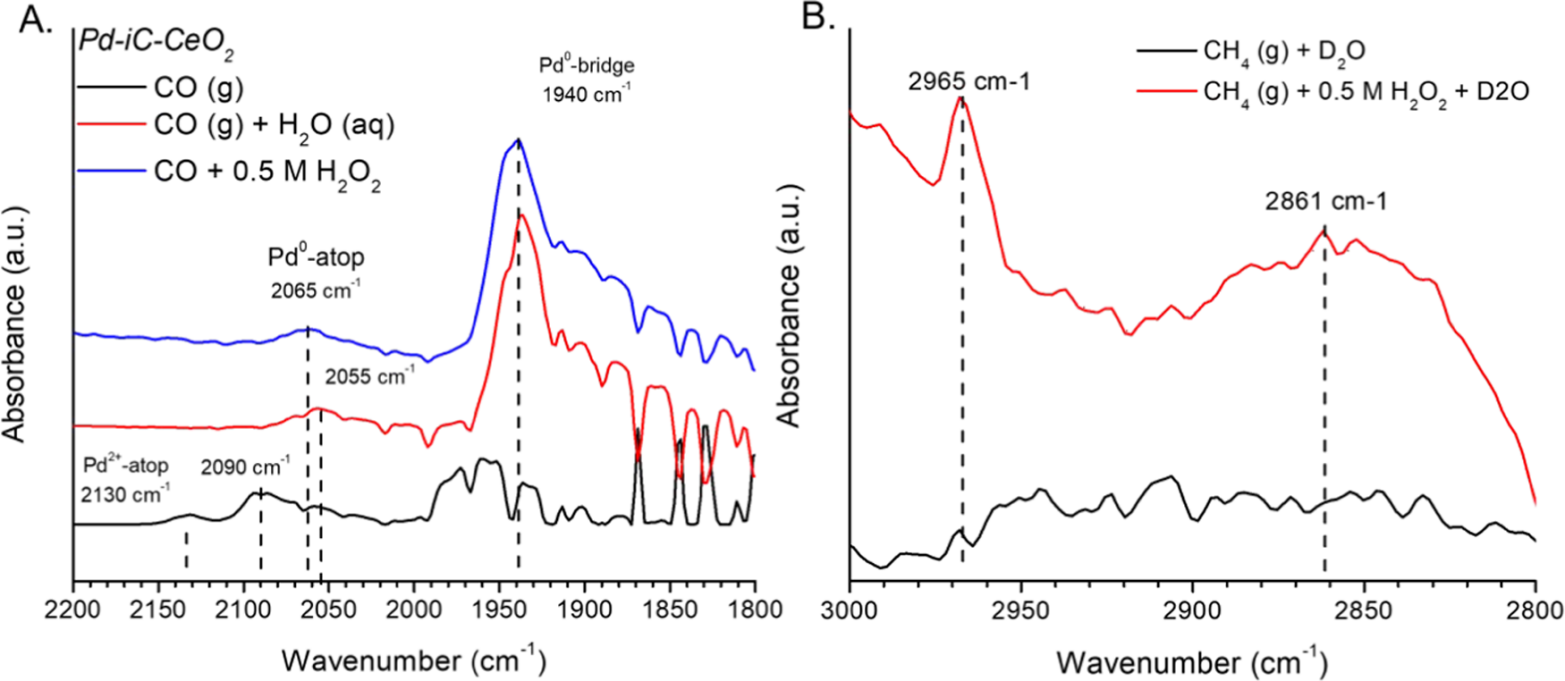
Influence of chemical environment on the surface structure of Pd-iC-CeO_2_. (A) CO-ATR of Pd-iC-CeO_2_ under pristine, aqueous, and 0.5 M H_2_O_2_ solutions (B) ATR deconvolution of individual MtM surface states at temperature after simulated reaction conditions in either pure D_2_O or 0.5 M H_2_O_2_/D_2_O conditions. Conditions: 10%CO/He or pure CH_4_ using 2 mL of D_2_O or 0.5 M H_2_O_2_ in a trough configuration measured in a static gas atmosphere simulating batch condition, conditions for reaction 10 mg catalyst suspended in either D_2_O, 0.5 M H_2_O_2_, or mixtures of D_2_O/H_2_O_2_, as indicated, and reacted in sealed ATR cell under 20 psig gas pressure for 1 h.

The pertinent surface chemistry of Pd-iC-CeO_2_ has been investigated through in situ attenuated total reflection infrared spectroscopy (ATR-IR) to explore reaction intermediates (Figure 6). Room temperature, ambient pressure CO-ATR spectra of Pd-iC-CeO_2_ were obtained in different environments: dry CO, a catalyst slurry in deionized water, and a catalyst slurry containing 0.5 M H_2_O_2_ (aq) in water (Figure 6A). In dry CO, the formation of highly dispersed Pd^2+^ species at 2130 cm^–1^, along with atop Pd^0^ sites at 2090 cm^–1^ and bridge sites at 1970 cm^–1^ is observed.^29^ However, in the presence of water and hydrogen peroxide, the Pd^2+^ atop sites transitioned to atop Pd^0^ at ∼2060 cm^–1^, and bridge sites shifted to 1940 cm^–1^. These changes are attributed to the solvent effects of water on the dipole moment of CO, a phenomenon typically observed in CO-ATR in contrast to gas phase diffuse reflectance infrared Fourier transform spectroscopy (DRIFTS) measurements.^30^ The formation of reduced Pd-CO species is attributed to the surface reduction as a result of exposure to CO, despite no reductive pretreatment before measurement and the catalysts being used as is after drop casting. This is consistent with the formation of bridge and hollow sites on ∼1.7 nm particles of Pd, even in the presence of a solvent (J. Cat., 2002, 210, 160–170). To isolate the effects of water and hydrogen peroxide, and remove contributions from broad OH bands, mixtures of D_2_O and D_2_O/H_2_O_2_ were used to access the MtM C–H reactive intermediates (Figure 6B). In the presence of the oxidant, H_2_O_2_, characteristic methoxy bands at 2965 and 2860 cm^–1^ appeared,^3,8,31^ reflecting successful conversion of methane into methanol. Notably, the catalyst shows methoxy formation even at the operating pressure of the ATR, which was approximately 20 psi CH_4_ partial pressure, which is in agreement with the findings from the catalytic reactor which operated at ∼4 bar (56 psi) CH_4_ partial pressure. In contrast, these bands did not appear in the case of CH_4_ + D_2_O alone, demonstrating that water alone is insufficient to activate the reaction due to the catalyst’s inability to dissociate D_2_O on its own. Furthermore, in the presence of 0.5 M H_2_O_2_ + D_2_O, the scrambling of H–O–H, H–O–D, and D–O–D bands at 1650, 1470, and 1290 cm^–1^, respectively (Figure S9), suggests that dissociated H_2_O_2_ rapidly complexes with deuterated water.^32^ This indicates that while water may not directly contribute to the methanol catalytic cycle, it plays an essential role in replenishing oxygen vacancies, recombining with peroxide, and providing surface OH sites. Hydrogen peroxide and peroxy radicals (OOH) are also observed at 3210 cm^–1^ and as a shoulder at 1420 cm^–1^ in the deuterated case, attributed to the unique cyclic recombination of HOOH–HOO species, which readily form complexes.^32,33^ CO-ATR following an in situ MtM reaction treatment at 75 °C (Figure S10) showed that both the presence of water and 0.5 M H_2_O_2_ (aq) did not affect the surface Pd adsorption sites. Atop and bridge Pd^0^ sites at 2051 and 1928 cm^–1^, respectively, were preserved relative to the room temperature CO-ATR. The shift of atop CO in 0.5 M H_2_O_2_ from 2060 to 2050 cm^–1^ is attributed to the decomposition of H_2_O_2_ after the reaction, where the H_2_O_2_ is rapidly consumed at 75 °C, where the absence dipole–dipole interactions between H_2_O_2_ derived radicals and CO, causes a shift to lower wavenumber after the H_2_O_2_ has been consumed,^30,32^ where post reaction acidified ceric sulfate titration has shown that the H_2_O_2_ is fully consumed upon completion of the reaction at 75 °C over Pd-iC-CeO_2_, where H_2_O_2_ decomposes into O_2_ and H_2_O. The preservation of the CO-Pd sites on the pristine material (Figure 6A) at room temperature versus after in situ reaction, measured at 75 °C (Figure S10), shows that the relevant active sites are preserved after reaction, consistent with the STEM imaging showing the Pd-iC-CeO_2_ moiety is preserved.

### Structural and Surface Analysis of Pd-iC-CeO_2_ via In Situ Methane to Methanol Conditions

**Figure 7:**
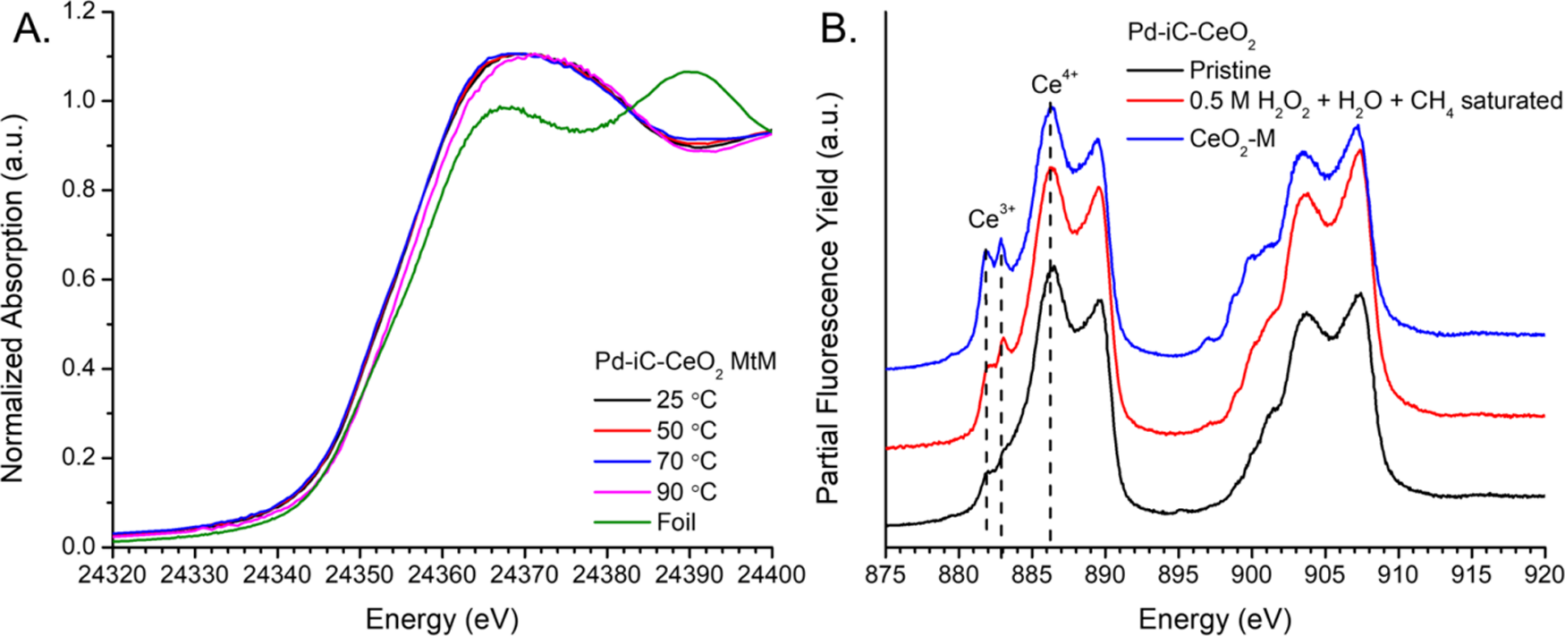
Formal oxidation state of Pd-iC-CeO_2_ in H_2_O_2_(aq) solvated conditions (A) Pd–K edge XAFS under high pressure methane to methanol conditions (34 bar total pressure, 20 bar CH_4_ pressure, 0.01 M H_2_O_2_ with 0.1 mL/min flow). (B) Ce M_4,5_-edge NEXAFS spectra comparing reference CeO_2_-M sample, pristine Pd-iC-CeO_2,_ and Pd-iC-CeO_2_ in the reaction solution measured using a static NEXAFS liquid cell charged with Pd-iC-CeO_2_ in 20 μL post reaction liquor after 1 h under MtM conditions.

In situ solvated X-ray absorption spectroscopy (XAS) and static solvated phase near edge X-ray absorption spectroscopy (NEXAFS) were carried out to determine the state of Pd and Ce, in the true reaction media of 0.5 M H_2_O_2_ (aq). The Pd K edge of Pd-iC-CeO_2_ (Figure 7A), measured in situ at the high-pressure liquid–gas–solid interface at 34 bar using a CH_4_ saturated (20 bar CH_4_ partial pressure) solution of 0.01 M H_2_O_2_ (aq), captures the true solvent effects of the aqueous reaction at pressures relevant to the reactor studies. The introduction of the reaction mixture shows that the system is stable up until 70 °C, beyond which the catalyst begins to be reduced toward a lower oxidation state, as evidenced by a shift in the white line at 90 °C. Based on linear combination analysis using pristine room temperature Pd-iC-CeO_2_ and Pd foil, the catalyst at 90 °C represents a 13% reduction toward a more reduced Pd state relative to the pristine material, while the other temperatures have no discernible difference with the pristine material. Under methane-saturated water in the absence of the oxidant, H_2_O_2_, Pd-iC-CeO_2_ did not experience a notable shifting of the white line as a function of temperature, only minor changes above the edge (Figures 7A and S11). However, upon addition of the oxidant, H_2_O_2_ (Figure S11A), there is a shift to a more oxidized state of Palladium which is reversed upon reaching 90 °C, mirroring the X-ray absorption near edge structure (XANES) of the system without oxidant (Figure S11B), likely due to the rapid decomposition of H_2_O_2_ that is also coupled to the onset formation of higher order oxygenates, i.e., CO_2_. Conversely, Pd-CeO_2_ exhibits no redox behavior during methane-to-methanol conversion in the presence of H_2_O_2_ or under the presence of CH_4_-saturated water at any temperature (Figure S12). Based on the findings from the Pd-iC-CeO_2_ XANES and the relative rates of peroxide decomposition of both systems this is likely due to the rapid decomposition of H_2_O_2_ over Pd-CeO_2_ which results in both systems appearing identical both with and without oxidant, as a mixture of O_2_ and H_2_O, the total H_2_O_2_ decomposition products, create a milder oxidizing environment than partial decomposition of H_2_O_2_ in the form of OH and OOH. XPS analysis indicates that the surface is primarily Pd^4+^, consistent with PdO_2_ species (Figure S13), in agreement with the Pd K edge showing a formal oxidation state >2. The minority species in the XPS, Pd^2+^, is likely an undercoordinated surface facing site that is ultimately reduced during Pd-CO ATR-IR to form an overlayer of *n* > 2 CO-Pd sites. However, XPS was measured under ex situ UHV conditions, while nominal exposures of CO to Pd can result in surface reduction even at low partial pressures of CO (Chem. Phys. Let. 1990, 5(6), 391–398), where CO-ATR was measured at 827 Torr CO (16 psi total pressure). The Pd 3d to C 1s ratio (Pd/C) of approximately 1:1.5 on the surface (Figure S13) shows that the carbon species retains its acetate-derived C_*x*_O_*y*_H features at 289 eV, enabling unique surface chemistry compared to inert C* species. Moreover, the C 1s spectrum shows that the acetate-derived features remain present even after the reaction (Figure S13). The combination of XAFS, XPS, and CO-ATR illustrate the need for various spectroscopic techniques to probe the multivalent surface/structure, showing the bulk properties, the reactive species which bind to CO and the subtle shift in surface structure under pristine conditions via XPS. The key findings from in situ XANES during MtM is that as the temperature is increased, the peroxide decomposes rapidly, resulting in the reduction of the Pd sites toward metallic Pd^0^ at 90 °C, which is correlated to the onset formation of CO_2_ to the presence of metallic Pd^0^ sites.

The influence of Ce was explored via Ce M_4,5_ Edge NEXAFS performed in a solvated static reaction cell to simulate batch reaction conditions (Figure 7B), which was measured at atmospheric pressure using a 20 μL aliquot of post reaction liquor and post reaction Pd-iC-CeO_2_ which was carried out in the high-pressure batch reactor under identical conditions as the kinetic measurements. Pure CeO_2_-M, milled CeO_2_ in the absence of Pd, shows lower-energy Ce^3+^ features at 882.0 and 882.9 eV^34^ due to the formation of defect sites during mechanochemical synthesis. The incorporation of Pd onto CeO_2_-M shows a decrease of the Ce^3+^ peaks, indicating that these defect sites are occupied by the intermediate Pd(OAc)_2_ precursor and eventual Pd-iC moiety. During in situ conditions, the Ce^3+^ peaks at ca. 882.0 and 882.9 eV suggest that defect sites, in addition to the Pd-iC-CeO_2_ moiety, are involved in the reaction, likely in the form of oxygen vacancies that can be readily saturated by the solvent., The NEXAFS Ce M_4,5_ was corroborated with post reaction XPS showing the Ce 3d spectra with primarily Ce^4+^ with a trace amount of Ce^3+^ (Figure S14). The evidence from NEXAFS and XAS supports the hypothesis that Pd-iC-CeO_2_ is modified by iC, which modulates the charge of Pd shown via charge distribution plots (Figure 5G,H), and ultimately dampens formation of CO_2_ and H_2_O_2_ decomposition, giving greater insights into the kinetic measurements by linking the increase in deep oxidation products (HCOOH, CO_2_) as a function of temperature to a reduction of Pd.

### DFT Modeling of Methane Activation and H_2_O_2_ Activation over Pd-iC-CeO_2_

Density functional theory (DFT) was employed to elucidate the nature of the active sites responsible for the exclusive selectivity toward methanol. Two distinct models were created: one involving the adsorption of a rhombohedral Pd_4_ cluster adsorbed on plain CeO_2_(111), denoted as Pd-CeO_2_ (Figure S15), and the other one created by adding four C–H species to Pd-CeO_2_ to represent the Pd-iC-CeO_2_ interface (Figures 5G and S15). C–H bonds were introduced into the model to stabilize the carbon species, recognizing that under experimental conditions, carbon is likely to be hydrogenated due to the presence of the solvent. In the Pd-CeO_2_ model, the four Pd atoms collectively transfer one e^–^, resulting in the reduction of one Ce^4+^ ion to a Ce^3+^ species. However, in the Pd-iC-CeO_2_ model, each of the four C atoms transfers one e^–^, leading to the formation of four Ce^3+^ species, while the Pd_4_ nanostructure, similar to the Pd-CeO_2_ case, donates another e^–^, forming a fifth Ce^3+^ species. The Bader charge analysis indicates that the Pd atoms are partially oxidized, as shown in Figure S15. The Pd/C:1 ratio used in the Pd-iC-CeO_2_ model catalyst in the DFT calculations closely matches the experimentally determined surface composition obtained via XPS. Test models with Pd nanoparticles approximately 1 nm in size have been calculated, and the activation of CH_4_ and H_2_O_2_ has been studied, yielding results similar to those obtained with the four-Pd models. This indicates that these models capture the essential physicochemical properties of the metal-(iC−) oxide interfaces.

The independent activation of H_2_O_2_ and CH_4_ was explored for both models (Figures S16–S17, respectively). These investigations revealed that, on Pd-iC-CeO_2_, the adsorption of H_2_O_2_ is approximately 0.8 eV weaker compared to Pd-CeO_2_, and the H_2_O_2_* → 2OH* decomposition reaction is 0.4 eV less exothermic (Figure S16), consistent with experimental work showing Pd-CeO_2_ has a 22% higher rate of decomposition than Pd-iC-CeO_2_. Furthermore, on Pd-iC-CeO_2_, the adsorption of CH_4_ is approximately 0.6 eV weaker compared to Pd-CeO_2_. Pd-CeO_2_ exhibits higher activity in cleaving of the first C–H bond in CH_4_*, with a 0.7 eV lower activation energy barrier compared to Pd-iC-CeO_2_ (Figure S17). Examining the geometry of the molecular states of H_2_O_2_* and CH_4_* on Pd-CeO_2_ and Pd-iC-CeO_2_, it is observed that the H–Pd and C–Pd distances in both molecular states are shorter in Pd-CeO_2_ compared to Pd-iC-CeO_2_ (Figures S16 and S17).

### Influence of the Carbon Interface on Ligand Effect and Strong Metal–Support Interaction (SMSI) in Pd-Based Catalysts

Our study demonstrates the significant influence of the carbon interface on the adsorption and activation processes of H_2_O_2_ and CH_4_ species on Pd-based catalysts, highlighting the roles of ligand effect and strong metal–support interaction (SMSI). Charge difference plots (Figures 5H and S18) further illustrate distinctions between the Pd-CeO_2_ and Pd-iC-CeO_2_ models. Analysis of the molecular states of H_2_O_2_* and CH_4_* on Pd-CeO_2_ and Pd-iC-CeO_2_ (see Figure S19), reveals notable differences in the H*–*Pd and C*–*Pd distances. In Pd-CeO_2_, these distances are shorter, facilitating easier molecule activation. This phenomenon is attributed to the ligand effect, where the CeO_2_ support withdraws electron density from Pd atoms, promoting an attractive molecule–surface interaction and facilitating bond cleavage, as previously reported.^33^ In the case of H_2_O_2_ adsorption, the O*–*Pd bond length is 202 pm for Pd-CeO_2_ and 219 pm for Pd-iC-CeO_2_ (Figure S19). This 17 pm bond length difference results in distinct charge transfer dynamics. In Pd-CeO_2_, charge predominantly transfers to an oxygen atom in the CeO_2_ support, with only 10% transferred to the H_2_O_2_ molecule, while the remainder goes to the oxide. In contrast, in the Pd-iC-CeO_2_ system, the entire charge transfers to the H_2_O_2_ molecule, highlighting the role of the carbon interface in facilitating complete charge transfer to the adsorbed molecule.

Similar geometrical trends are observed in the adsorbed state of CH_4_ (Figure S19g–j). In Pd-CeO_2_, there is a noticeable 6 pm elongation in the C*–*H bond, whereas in Pd-iC-CeO_2_, this elongation is only 1 pm. Charge analysis reveals that CH_4_* donates approximately 0.1e^–^ to Pd-CeO_2_, which is accepted by the oxide support. In contrast, on Pd-iC-CeO_2_, only 0.03 are donated, with the charge is entirely transferring to the Pd nanoparticle, indicating that the carbon interface acts as a barrier for charge transfer to the oxide. Moreover, the d_*z*^2^_-projected density of states on the Pd atom, where CH_4_ adsorbs, reveals an occupancy of 71.5% in Pd-CeO_2_ and 98.2% in Pd-iC-CeO_2_ (Figure S20). The lower d_*z*^2^_ occupation in the Pd-CeO_2_ is attributed to ligand effects, highlighting that the CeO_2_ support withdraws electron density from the Pd atoms.

Finally, in the case of 2OH groups adsorbed on the Pd nanoparticles, charge analysis indicates that the Pd nanoparticle in Pd-CeO_2_ donates 0.87e^–^, whereas in Pd-iC-CeO_2_, it donates 0.97e^–^. Nearly the entire charge is transferred to the 2OH groups, resulting in excess charges of 0.85 and 0.94e^–^, respectively. This behavior is primarily due to the OH^–^ groups’ tendency to capturing charge.

The carbon interface in Pd-iC-CeO_2_ restricts charge transfer to the CeO_2_, thereby modulating the SMSI. This modulation reduces the interaction between Pd and CeO_2_, allowing the Pd nanoparticles to retain more charge and enhancing the overall catalytic performance. Overall, these findings underscore the crucial role of the carbon interface in influencing adsorption geometries, activation energies of reactive species, and charge transfer processes on Pd-based catalysts

### Langmuir–Hinshelwood Approximation for Methane to Methanol

**Figure 8:**
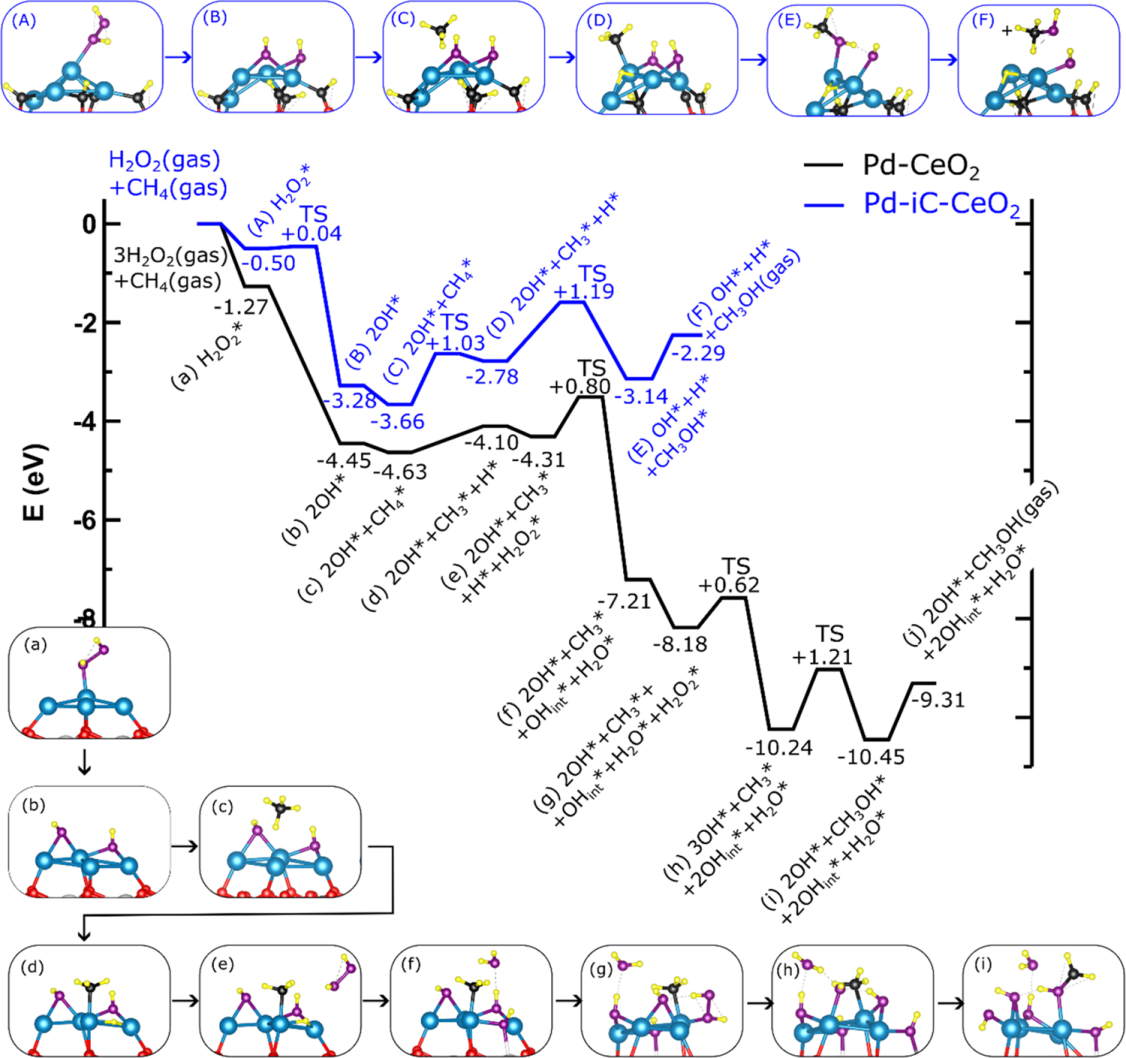
Mechanisms for methanol formation on Pd-iC-CeO_2_ (A–F, blue path) and Pd-CeO_2_ (a–i, black path) catalysts. The same color coding is used as in Figure 5, additionally the O atoms from the H_2_O_2_ species are denoted in violet.

The comprehensive MtM reaction mechanism is compared for both models in Figure 8, following a traditional gas-phase Langmuir–Hinshelwood (LH) mechanism for both Pd-CeO_2_ and Pd-iC-CeO_2_. The analysis reveals that surface OH* species mainly affect the stabilization of CH_4_* species. On Pd-iC-CeO_2_, the binding of CH_4_* species is 0.2 eV stronger than on Pd-CeO_2_ (Figure 8). When OH groups are coadsorbed on Pd-iC-CeO_2_, the CH_4_ loses 0.01e of charge compared to the case of CH_4_/Pd-iC-CeO_2_ without OH groups. This charge is transferred to the OH groups, leading to increased adsorption energy for CH_4_ species when coadsorbed with OH species. Conversely, in the (CH_4_ + 2OH)/Pd-CeO_2_ system, CH_4_ gains 0.07e^–^ compared to the CH_4_/Pd-CeO_2_ case without OH groups. The charge on the OH groups and the Pd nanoparticle remains unchanged. This indicates that the charge gained by CH_4_ is transferred by the CeO_2_ support. Therefore, as CH_4_* becomes more charged, the adsorption energy decreases. Additional analysis further confirms that on both Pd-CeO_2_ and Pd-iC-CeO_2_ surfaces, the rate-limiting step is the formation of CH_3_OH* (*E*_act_ = 1.21 and 1.19 eV, respectively). The presence of carbon may act as passivating agent for Pd, resulting in reduced overoxidation, similar to the effect observed for Cu–Ag interfaces, where the presence of a unique bimetallic site was necessary to favor liquid oxygenate yield.^18^ Notably, in the case of Pd-iC-CeO_2_, CH_3_OH* is formed from CH_3_* and OH* species immediately following the dissociation of CH_4_* (Figure 8). In contrast, the same step on Pd-CeO_2_ would have a higher activation barrier of 1.53 eV (Figure S21). However, this barrier can be reduced by adding H_2_O_2_ molecules, where Pd-CeO_2_ consumes three H_2_O_2_ molecules (Figures 8 and S21–S22) while Pd-iC-CeO_2_ only requires one H_2_O_2_ molecule to drive the reaction forward (Figures S23–S24). This comparison reveals that the presence of carbon at the interface of the hydroxylated surface has a dual effect: it favors the stabilization of the methane but also blocks sites where highly oxidizing OH_int_ species form. This is notably in agreement with the experimental reduced rate of H_2_O_2_ decomposition.

### Modeling Solvent Effects via Eley–Rideal-like Mechanism on Pd-Based Catalysts

The effect of hydrogen peroxide in an aqueous medium was also considered to analyze an Eley–Rideal-type mechanism, in which CH_3_OH is formed through the interaction of CH_3_* with OH from the aqueous phase. The initial-state structures chosen for this study are shown in Figure S25. For Pd-iC-CeO_2_, the starting point is adsorbed CH_3_* + H* + 2OH* (states shown in Figures 8D and S25), whereas for Pd-CeO_2_, we begin with CH_3_* + 2OH* + OH_int_* (state in Figure 8f without the H_2_O* molecule). The selection of different degrees of hydroxylation was based on the fact that Pd-CeO_2_ was more prone to hydroxylation.

**Figure 9:**
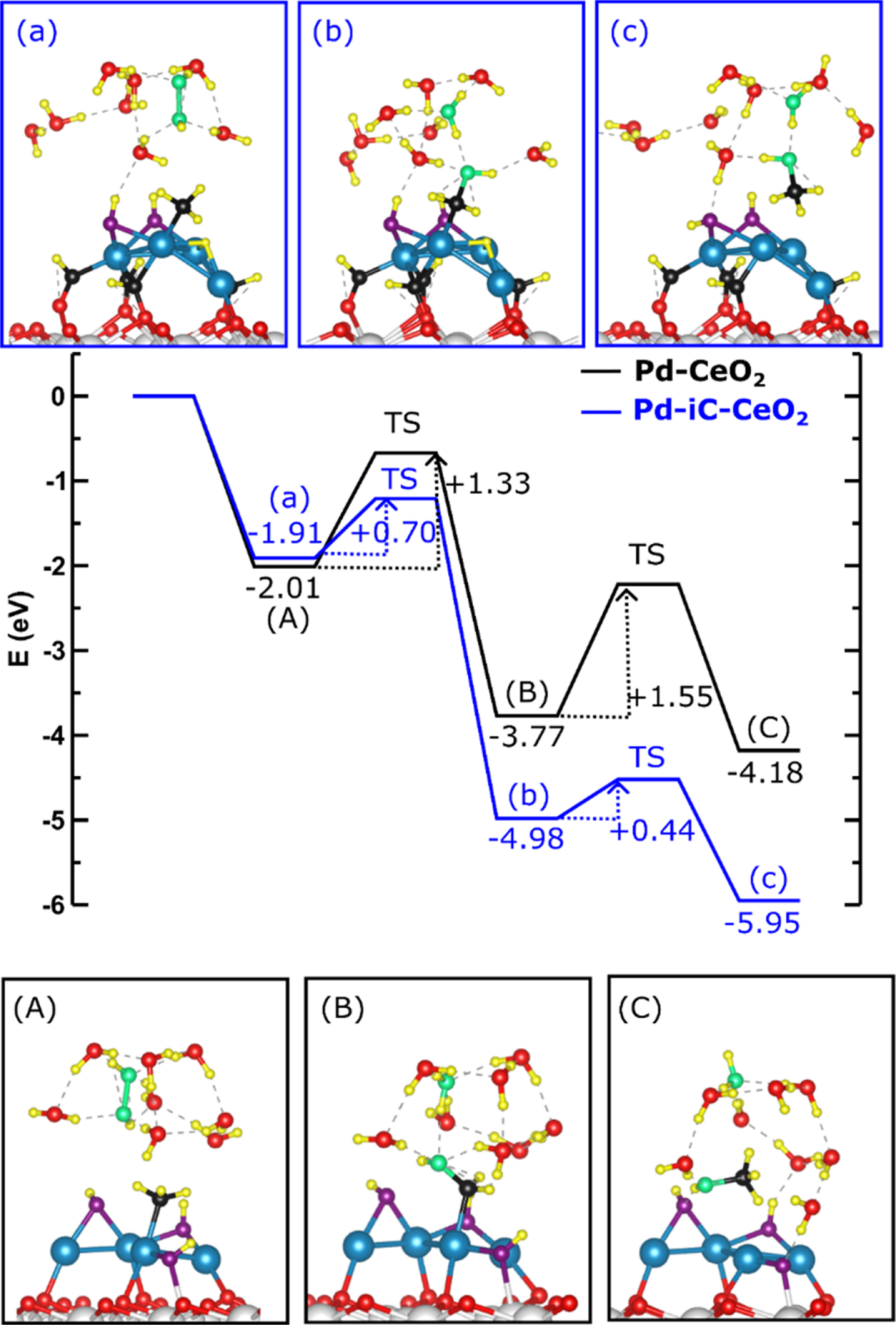
Eley–Rideal type mechanism for the formation of CH_3_OH on Pd-CeO_2_ (A–C, black path) and Pd-iC-CeO_2_ (a–c, blue path). The first step of the mechanism involves the formation of CH_2_OH and then the formation of CH_3_OH. The adsorption energy of each stable state and the activation energy associated with the transition states relative to their precursor states are included.

Hydrogen peroxide in an aqueous medium was modeled by including the 8H_2_O·2OH(aq) complex, as shown in Figure S25. In the case of Pd-iC-CeO_2_, this aqueous complex was adsorbed onto the previous model (CH_3_* + 2OH* + H*)/Pd-iC state (resulting in new states, as shown in Figures 9a and S26). As can be seen, 2OH eventually formed H_2_O_2_. Then, H_2_O_2_(aq) is activated to 2OH(aq), where one OH(aq) breaks the C*–*Pd bond, forming an unstable CH_3_OH. The C*–*H and O*–*H bonds are then broken, where one H binds to the Pd nanoparticle and the other H forms an H_2_O molecule with the remaining OH(aq), leaving an unstable CH_2_O* species. Finally, the H atom initially bound to the Pd nanoparticle binds to the O atom of CH_2_O, forming CH_2_OH*, with an overall barrier of 0.70 eV (see Figure S26 top panel). In contrast, the formation of CH_2_OH* on Pd-CeO_2_ with the 8H_2_O·2OH(aq) moiety results in a barrier of 1.33 eV (Figure 9), which includes the formation of 2OH species from H_2_O_2_, formation of CH_3_ and the corresponding H* abstraction, and the final CH_2_OH formation. This significant difference is due to the different pathways, as explained, where the carbon atom in CH_3_* + 2OH* + H* on Pd-iC-CeO_2_ (see Figure 9a) is 0.09e more charged than that in CH_3_* + 2OH* + OH_int_* on Pd-CeO_2_ (see Figure 9A). This results in a C*–*Pd bond length of 211 pm in Pd-iC-CeO_2_ compared to 202 pm in Pd-CeO_2_. This difference makes CH_3_* on Pd-iC-CeO_2_ more labile to C*–*Pd bond breaking, leading to distinct pathways for CH_2_OH formation on each catalyst. Consequently, on Pd-iC-CeO_2_, OH(aq) breaks the C*–*Pd bond, forming unstable CH_3_OH species, whereas on Pd-CeO_2_, the C*–*H bond breaks, forming a CH_2_ species that is labile to CH_2_OH formation. Notably, the CH_2_OH species is by 1.21 eV more stable on the Pd-iC-CeO_2_ catalyst than on Pd-CeO_2_. Furthermore, the formation of CH_2_OH on Pd-iC-CeO_2_ yields an energy gain of 3.07 eV, whereas on the energy gain Pd-CeO_2_ is only 1.76 eV, which is 1.31 eV less.

The last step of the mechanism involves the formation of CH_3_OH from CH_2_OH, with differences between the two models. In Pd-CeO_2_, the hydrogen atom comes from OH_int_*, whereas in Pd-iC-CeO_2_, it comes from the adsorbed H* (see Figures 9 and S27). This difference is due to the Pd-CeO_2_ model catalyst being more hydroxylated than Pd-iC-CeO_2_, increasing the probability of H* interacting with OH species to form H_2_O* and thus decreasing the H* concentration. Breaking the O*–*H bond is more difficult and leads to a high activation barrier for CH_3_OH* formation, *E*_act_ = 1.55 eV, which is much higher than that of Pd-iC-CeO_2_, *E*_act_ = 0.44 eV.

From these results, it can be concluded that in the case of Pd-iC-CeO_2_, the activation barriers are low, and the limiting step is the initial CH_4_ activation, requiring 1.03 eV (Figure 9). In the case of Pd-CeO_2_, CH_4_ activation is facile (0.33 eV), and the highest barriers are found for the formation of CH_2_OH and CH_3_OH (1.33 and 1.55 eV). This suggests that the limiting step for Pd-CeO_2_ is the formation of CH_3_OH.

### Balancing LH and ER Mechanisms for Direct Methane to Methanol Conversion

In the final step of the MtM reaction, the desorption of CH_3_OH plays a critical role. In the LH mechanism (Figure 8), CH_3_OH* desorbs with energies of 1.14 and 0.85 eV from the Pd-CeO_2_ and Pd-iC-CeO_2_ surfaces, respectively, which is associated with the cleavage of a Pd–O bond of 2.08 and 2.19 Å, respectively. However, in the ER mechanism (Figures 9 and S25), the formed CH_3_OH(aq) species are already detached from the surfaces, with Pd–O distances of 2.57 and 2.72 Å for the Pd-CeO_2_ and Pd-iC-CeO_2_ surfaces, respectively. This is a result of the occupancy of the filled states on the Pd site of Pd-iC-CeO_2_ as discussed above. Consequently, the presence of the Pd-iC-CeO_2_ interface significantly facilitates methanol desorption, and the role of solvation effects is crucial for its effortless removal. This underscores that while water may not be a main reactive species by itself, its function as a carrier for the catalytically relevant OH species is paramount in driving the selective conversion of methane to methanol. These findings align with the existing literature on gas-phase methane to methanol conversion, which highlights the need for water vapor flow to desorb the methanol species. The synergistic effect arising from the presence of carbon at the Pd-iC-CeO_2_ interface, and the influence of solvation effects are the key factors that render the Pd-iC-CeO_2_ catalysts highly active and selective for the MtM reaction.

## Conclusions

In summary, this study underscores the pivotal role of modulating the activity of highly active metal-oxide interfaces by the incorporation of carbon interlayers. Particularly in partial oxidation reactions, where the formation of undesirable total oxidation products is a challenge, precise control over the activation of the oxidant and the reactant emerges as a critical factor for achieving highly selective partial oxidation outcomes. Interfacial carbon proves essential for balancing the activation of the oxidant in tandem with the CH_4_ to achieve ideal product selectivity. These findings hold the promise of paving the way for cost-effective metal–carbon-oxide catalysts that harness the synergistic properties of reducible oxide supports and highly active metal centers while leveraging unique solvent effects to enhance the production of desired target products. This breakthrough opens new avenues for advancing catalytic process, contributing to sustainable and efficient chemical transformations, and ultimately fostering cleaner and more environmentally friendly chemical industries in the future.

## Experimental Methods

### Catalyst Synthesis

The catalysts were synthesized following dry mechanochemical synthesis, outlined in detail elsewhere.^22^ Briefly, Palladium(II) acetate (Sigma-Aldrich, 99.99%) was mixed with a corresponding amount of a commercial ceria support (Rhodia), previously calcined at 900 °C for 3 h (Brunauer–Emmett–Teller (BET) surface area ≈25 m^2^/g). The resulting catalyst obtained after the mechanochemical synthesis, referred to as Pd-iC-CeO_2_, was used as is, unless otherwise specified. Comparative catalysts were prepared by conventional incipient wetness impregnation,^21^ Pd-CeO_2_, and by milling Pd acetate over SiO_2_ previously calcined at 800 °C for 3 h (BET surface area 5 m^2^/g), following the same milling procedure,^21^ and denoted as Pd-SiO_2_. All samples have a final nominal Palladium loading of 4 wt %.

### Catalytic Performance Evaluation

The methane to methanol experiments were carried out using a commercial Parr autoclave reactor (Parr 5500 Reactor). Typically, 25 mg of catalyst was added to 15 mL of 0.5 M H_2_O_2_ aqueous solution and stirred at 800 rpm. The sealed solution was flushed with 20% CH_4_ balanced in Ar gas mixture 5 times to remove residual air contaminants and then pressurized to 20 bar under the same 20% CH_4_ mixture gas (4 bar CH_4_, 16 bar Ar). The head gas was continuously analyzed using an online gas chromatography (GC) equipped with TCD/FID (Agilent 7890B) detectors connected directly to the Parr reactor for the analysis of methane and CO_2_ before and after the reaction. The reaction solution was rapidly heated to the desired temperature and held for the specified time. Subsequently, the entire Parr reactor was quenched in an ice bath (<10 °C) to prevent further reaction and the volatilization of oxygenate products via flashing. Liquid products were analyzed via ^1^H NMR (Bruker AVANCE 400 MHz) using 0.1 mL of 0.1 wt % 3-(trimethylsilyl)-1-propanesulfonic acid-*d*_6_ sodium salt (DSS) prepared in D_2_O as the locking agent (DSS/D_2_O) as an internal standard, along with 0.7 mL of the filtered reactor solution. The ^1^H NMR was quantified by calibrating against known standards of the oxygenates relative to the DSS peak. Samples with pretreatment were prepared ex situ in a tubular furnace using polished quartz crucibles before charging into the batch reactor. All measurements were performed in triplicate, as batch reactor systems can introduce higher error than traditional gas-phase reactors due to additional preparation steps. Recycle runs from methane to methanol were performed sequentially, with the reactor solution being evaluated after each run. Finally, the catalysts were washed, recovered, and dried in air, where the subsequent cycles were carried out following the same procedure listed above for a typical reaction. Hydrogen peroxide concentrations were determined via acidified ceric sulfate titration, where the relatives rates of peroxide decomposition were carried out at 25 °C and 1 atm pressure under ambient air using 15 mL of ∼0.5 M H_2_O_2_ and 25 mg of catalyst under mixing. Blank H_2_O_2_ was the relative rate of decomposition of the pure aqueous peroxide mixture in the absence of a catalyst at standard temperature and pressure.

### Powder X-ray Diffraction (pXRD)

Powder XRD patterns were collected using a Philips X′Pert Diffractometer equipped with an X′Celerator detector, using Ni-filtered Cu Kα radiation (λ = 1.542 Å) operating at 40 kV and 40 mA. Diffractograms were collected in the 20–80° 2θ range, with 0.02° step size and 40 s counting time per step.

### Thermogravimetric Analysis (TGA)

TGA experiments were carried out in a Q500 thermogravimetric apparatus (TA Instruments), loading ca. 15 mg of sample in a platinum pan and increasing the temperature at 10 °C/min up to 600 °C under 60 mL/min of air.

### Attenuated Total Reflectance Infrared Spectroscopy (ATR-IR)

The ATR-IR measurements were conducted on a Bruker Vertex 70 Infrared Bench using a commercial Harrick Horizon Multiple Reflectance ATR cell equipped with a special high pressure and temperature liquid cell, which was constructed from Hastelloy (generic name, Alloy C-276) in all wetted parts to prevent cell degradation under peroxide conditions and adapted for high temperature and pressure, shown in detail in Figure S28. The cell was connected to an MCT detector with a resolution of 4 cm^–1^. A Si ATR 45° prism was used due to its chemical inertness, as ZnSe decomposes under solvated peroxide conditions. For dry measurements, the catalyst was dropcast directly onto the Si ATR prism using a slurry of catalyst and deionized (DI) water. Gas (He, CO, or CH_4_, all UHP) was flowed over the catalyst at the specified temperature at a total flow rate of 20 mL/min. Solvated liquid phase ATR measurements were performed by preparing a slurry of the catalyst in the specified solvent (H_2_O, D_2_O, 0.5 M H_2_O_2_ (aq), 0.5 M H_2_O_2_ in D_2_O). This was achieved by dispersing the finely ground catalyst and sonicating the solution for approximately 10 min. The resulting slurry was loaded into the Horizon Cell trough (approximately 1 mL of slurry) and purged with He to remove residual air contaminants. The background of all measurements was taken after He flushing at the specified temperature. For CO-ATR 10%CO/He was used for all CO-ATR measurements in either DI H_2_O, 0.5 M H_2_O_2_ (aq), while UHP CH_4_ was used for the simulated methane to methanol conditions using either a mixture of 0.5 M H_2_O_2_ prepared in H_2_O or D_2_O, as listed, or purely CH_4_ in either H_2_O or D_2_O. In the case of methane to methanol ATR measurements, the cell was first purged with CH_4_ to remove residual inert gas and saturate the slurry. Subsequently, it was sealed and pressurized to approximately ∼20 psi of CH_4_, and measurements were taken as a function of time. The process was completed with a desorption step in He. The catalyst bed remained wetted throughout the entirety of the experiments, evidenced by remaining liquid upon removing the crystal and sample.

### Scanning Transmission Electron Microscopy (STEM)

STEM imaging and STEM-EELS were performed using Hitachi HD2700C dedicated STEM with the probe Cs corrector and Gatan Enfinium EELS at an accelerating voltage of 200 kV in the Center for Functional Nanomaterials at Brookhaven National Laboratory. All STEM images were acquired with the secondary electron (SE) detector to clearly image Pd nanoparticles on ceria support.

### Near Edge X-ray Absorption Spectroscopy (NEXAFS)

NEXAFS measurements were carried out at the National Synchrotron Light Source II (NSLS-II) at Brookhaven National Laboratory at the In situ and Operando soft X-ray spectroscopy (IOS) beamline, 23-ID-2. Ex situ measurements were done by dropcasting the powders onto Indium Foil strips prior to loading into the UHV endstation, where the C and O K-edges were measured in addition to the Ce M_5/4_-edge. In situ measurements were carried out using a custom-built static liquid cell sealed with a 100 nm-thick X-ray transparent Si_3_N_4_ window. The cell was charged with the catalyst extracted from the Parr reactor at the following operating conditions: 20 bar pressure (20% CH_4_), 75 °C, 800 rpm, 15 mL of 0.5 M H_2_O_2_ (aq), and 25 mg of catalyst, with a 1 h reaction time. The resulting slurry was dropcast onto the Si_3_N_4_ membrane of the cell to allow for intimate contact with the window while 19 μL of the reaction liquor was charged in the static liquid reservoir to replicate solvent effects. Partial fluorescence yield spectra were acquired using a Vortex EM silicon drift detector.

### X-ray Photoemission Spectroscopy

A commercial SPECS AP-XPS chamber equipped with a PHOIBOS 150 EP MCD-9 analyzer in the Chemistry Division of Brookhaven National Laboratory was used for XPS measurements. For energy calibration, the Ce 3d photoemission line with the strongest Ce^4+^ feature at 916.9 eV, was used. The powder catalysts were drop cast onto a roughened aluminum plate and then loaded into the AP-XPS chamber. All XPS measurements were conducted at room temperature under UHV conditions using an Al Kα X-ray anode (1486.6 eV) where the pass energy was 50 eV and averaged over 15 scans with a dwell time of 0.1 s.

### X-ray Absorption Spectroscopy (XAS)

In situ XAS spectra were collected at the Inner Shell Spectroscopy beamline 8-ID at the National Synchrotron Light Source II in Brookhaven National Laboratory. The Pd K-edge was collected under in situ conditions using a custom-made high pressure flow reactor, shown in Figure S29 which was constructed from 316 stainless steel. XAS spectra were recorded in fluorescence mode using a PIPS detector. Pd foil was measured for energy calibration, using ion chambers, by moving the reaction cell out of the beam path. A CH_4_ saturated flow of a 0.01 M solution of H_2_O_2_ with a rate of 0.1 mL/min was introduced to a custom-made reaction cell with a fixed catalyst bed placed in between two quartz wool plugs. The flow was controlled using a high-pressure compact pump (Azura, Knauer), the pressure in the reaction cell was maintained using a 34 bar back-pressure regulator (UpChurch) in addition to the use of graphite windows to withstand the cell pressure while accommodating the beam, while the tests were carried out at 50, 70, and 90 °C. To determine the redox properties of the materials prior to MtM reaction a H_2_O solution saturated with CH_4_ was introduced into the cell (34 bar, 50, 70, and 90 °C), denoted as simply CH_4_ in the relevant figures.

### In Situ ^1^H Nuclear Magnetic Resonance (NMR)

In situ ^1^H NMR experiments were collected on a Bruker 700 MHz spectrometer, equipped with a *z*-shielded gradient triple resonance 5 mm TCI cryoprobe. Temperature calibration was achieved using ethylene glycol. Each transient spectrum was acquired with 64 scans and a relaxation delay of 2 s. The probe was preheated to the desired temperature (75 or 90 °C) before loading the high-pressure NMR tube (SP Industries Co., 524-PV-7, 5 mm O.D. and 7 in. length) into the spectrometer. The spectra acquisition commenced after temperature equilibration for ca. 13–14 min. The NMR tube was loaded with Pd-iC-CeO_2_ (2.3 mg) and 0.5 M H_2_O_2_ in H_2_O/D_2_O solution (3% H_2_O; 97% D_2_O; 400 μL), pressurized with 5 bar methane. The reaction constants were calculated by curve-fitting the time-resolved [MeOH] profile using pseudo-first-order rate equation: [MeOH] = *a* × exp(−*k* × *t*) + *b*.

### Density Functional Theory (DFT) Calculations

The calculations were performed using density functional theory (DFT) as implemented in VASP code (version 6.3.0),^35^ which uses the slab-supercell approach.^36^ The projector augmented wave (PAW) method^37^ was used to describe the valence electrons of the atomic species: Ce (4f, 5s, 5p, 5d, 6s), O (2s, 2p), Pd(4p, 4d, 5s), C(2s, 2p) and H (1s), with a plane-wave cutoff energy of 415 eV. Electron localization due to electron transfer from the metal (or C species) to the oxide support, has been treated by means of the DFT + *U* approach proposed by Dudarev et al.,^38^ with a *U*_eff_ value of *U* – *J* = 4.5 eV for the Ce 4f electrons. Additionally, the generalized gradient approximation (GGA), as suggested by Perdew, Burke, and Ernzerhof (PBE) was used.^39^ Long-range dispersion corrections were considered with DFT lattice constants, employing the so-called DFT-D3 approach.^40,41^

The CeO_2_(111) surface with (3 × 3) periodicity was modeled with an optimized lattice constant of 5.485 Å for bulk CeO_2_. All surface models used in this work have two O–Ce–O trilayers slabs and ∼21 Å of vacuum separation between consecutive slabs. All atoms in the bottom O–Ce–O trilayer were kept fixed at their optimized bulk-truncated positions during geometry optimization, whereas the rest of the atoms were allowed to fully relax. A (2 × 2 × 1) *k*-point mesh, according to the Monkhorst–Pack method, is used to sample the Brillouin zone.^41,42^ To create the Pd-CeO_2_ and Pd-iC-CeO_2_ models, rhombohedral planar Pd_4_ clusters were considered. In the case of Pd-CeO_2_, this cluster is in direct contact with the ceria support, and the four Pd atoms collectively transfer one e^–^, resulting in the reduction of one Ce^4+^ ion to Ce^3+^ species (see Figure 5G). In contrast, the Pd-iC-CeO_2_ model was created by inserting four (C–H) species below the rhombohedral Pd_4_ structure forming Pd–C–O bonds. After the structure is optimized, it is observed that only one Pd atom forms a bond with the oxidic support (see Figure 5H) and that each of the four C atoms transfers one e^–^, leading to the formation of four Ce^3+^ species. Additionally, the Pd_4_ nanostructure, similar to the Pd-CeO_2_ case, donates another e^–^, forming a fifth Ce^3+^ species. The charge analysis involved calculating Bader charges on Pd and estimating the oxidation state of Ce atoms based on their local magnetic moment. This magnetic moment, representing the difference between up and down spin on the atoms, was obtained by integrating the site- and angular momentum projected spin-resolved density of states over spheres with radii chosen as the Wigner–Seitz radii of the PAW potentials. For reduced Ce ions, the occupation of Ce f states is close to 1, and the magnetic moment is ∼1 μB. Therefore, these ions are referred to as Ce^3+^. It is important to note that the inclusion of H-bonded C species prevents the formation of oxygen vacancies through the formation of CO species, which would result from the removal of lattice oxygen. For the gas-phase calculations of the CH_4_, H_2_O_2_, and H_2_O molecules, a (15 × 14 × 13) Å^3^ cell was employed, with Γ-point only.

The (co)adsorption energies of methane, hydrogen peroxide, and the 8H_2_O·2OH(aq) complex were calculated according to the following equation
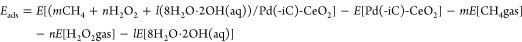
where *E*[(*m*CH_4_ + *n*H_2_O_2_ + *l*H_2_O)/Pd(-iC)-CeO_2_] is the total energy of *m* methane, *n* hydrogen peroxide and *l* water molecules (co)adsorbed on the surface with *n* = 0, 1, *m* = 0–3, *l* = 0, 1, *E*[Pd(-iC)-CeO_2_] is the total energy of the clean model catalyst: Pd-CeO_2_ or Pd-iC-CeO_2_, *E*[CH_4_gas] and *E*[H_2_O_2_gas] are the energies of the methane, hydrogen peroxide and water molecules in the gas phase. *E*[8H_2_O·2OH(aq)] is the energy of the 8H_2_O·2OH(aq) complex far from the surface.

To identify transition state (TS) structures, the climbing image nudged elastic band technique (CI-NEB) was used.^43^ Among all the TSs discussed in this study, a sole imaginary frequency has been identified. Conducting complete geometry optimizations starting from the nearest configurations behind and ahead (along the reaction path) of this TS leads to a nondissociated state and a dissociated state, respectively. Within the computed potential energy profiles, the activation energy (*E*_act_), defined as the difference between the energy of the transition state (ETS) and the initial state (EIS), serves as an indicator of the activation energy.

## Data Availability

The DFT data that support the findings of this study are available in Materials Cloud {https://www.materialscloud.org/home} with the identifier DOI: 10.24435/materialscloud:dz-zz. The data is also available from the authors upon reasonable request.

## Supporting Information Available

Experimental results: Detailed methane to methanol product distribution, characterization of the material (diffraction, XANES, XPS, ATR-IR, TGA, STEM-EELS), dispersion calculations, and schematics for high pressure gas–solid–liquid characterization cells. Theoretical results: Expanded discussion of the mechanism of methanol formation on Pd-CeO_2_, hydrogen peroxide decomposition, charge density difference plots (CDDP) and density of states (PDOS) during the reaction, and additional justification of the Langmuir–Hinshelwood (PDF)
Eley–Rideal Pd-CeO_2_ (MP4)
Eley–Rideal Pd-iC-CeO_2_ (MP4)
