# Supplementary material for: From Methane to Methanol: Pd-iC-CeO2 Catalysts Engineered for High Selectivity via Mechanochemical Synthesis
Source: J Am Chem Soc. 2024 Aug 15;146(38):25986–99. doi: 10.1021/jacs.4c04815 (PMC11440493; doi:10.1021/jacs.4c04815)
Supplement: Supplementary file 1 — ja4c04815_si_001.pdf [file ja4c04815_si_001.pdf]

Supplemental Information

From Methane to Methanol: Pd-iC-CeO_2 Catalysts Engineered for High Selectivity via Mechano-Chemical Synthesis

Juan D. Jiménez[1], Pablo G. Lustemberg[2*], Maila Danielis[3], Estefanía Fernández-Villanueva[2,4], Sooyeon Hwang[5], Iradwikanari Waluyo[6], Adrian Hunt[6], Dominik Wierzbicki[6], Jie Zhang[7], Long Qi[7], Alessandro Trovarelli[3], José A. Rodriguez[1,8], Sara Colussi[3], M. Verónica Ganduglia-Pirovano[2*], and Sanjaya D. Senanayake[1*]

[1] Chemistry Division, Brookhaven National Laboratory, Upton, NY, 11973, USA

[2] Instituto de Catálisis y Petroleoquímica, CSIC, C/Marie Curie 2, 28049 Madrid, Spain

[3] Polytechnic Department, University of Udine and INSTM, Via del Cotonificio 108, 33100 Udine, Italy

[4] Universitat Politècnica de València, Camí de Vera s/n, 46022, València, Spain

[5] Center for Functional Nanomaterials, Brookhaven National Laboratory, Upton, NY, 11973, USA

[6] National Synchrotron Light Source II, Brookhaven National Laboratory, Upton, New York 11973, USA

[7] Ames National Laboratory, Iowa State University, Ames, Iowa, 50011, USA

[8] Department of Chemistry, State University of New York Stony Brook, Stony Brook, NY 11794, USA

*Corresponding Email: p.lustemberg@csic.es

*Corresponding Email: vgp@icp.csic.es

*Corresponding Email: ssenanay@bnl.gov

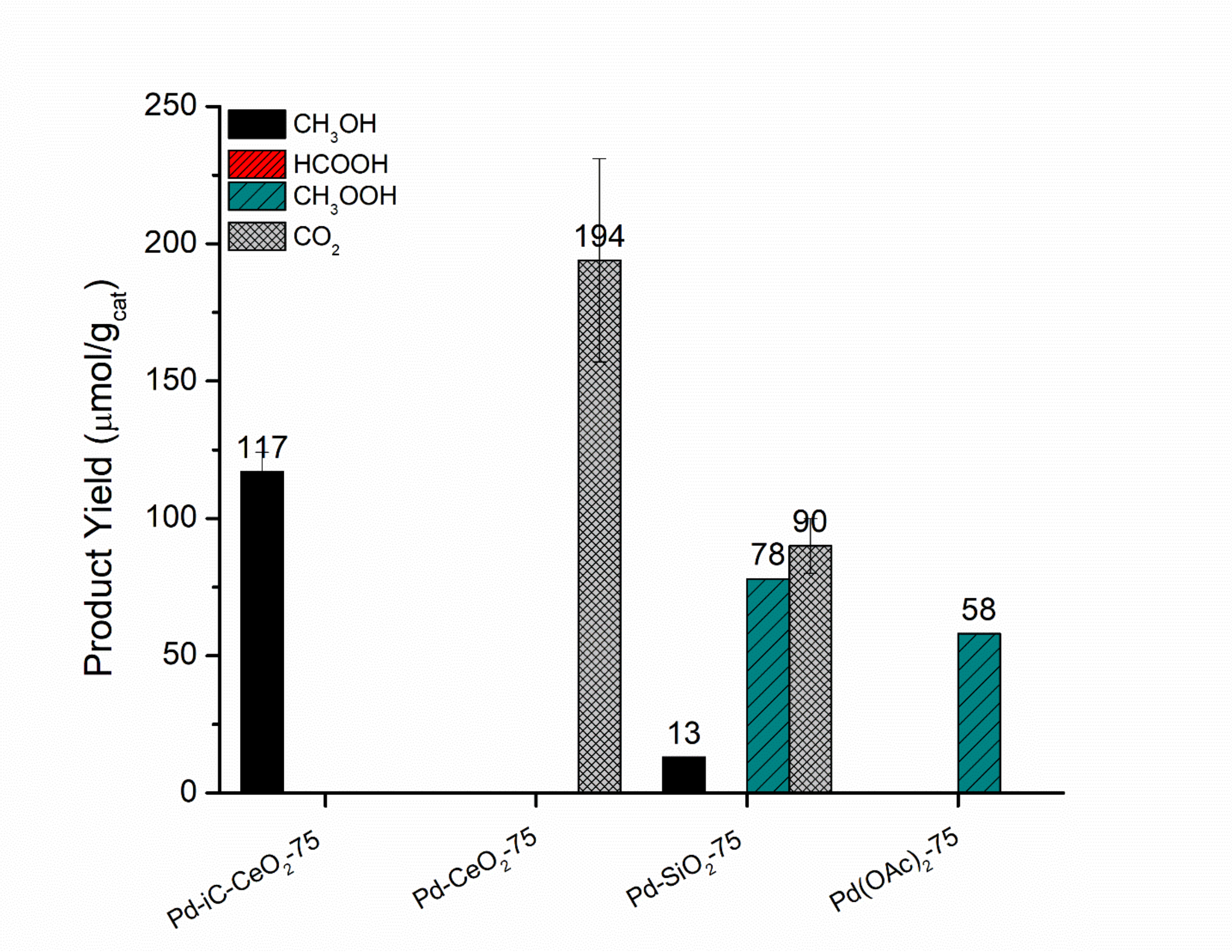

Figure S1: Methane to methanol product distribution for various Pd reference materials. Conditions: 75 °C reaction temperature, 15 mL 0.5M H_2O_2 (aq) solution, 25 mg catalyst (1 mg equivalent Pd loading), 800 rpm, 20 bar total initial pressure (20% CH_4, balance Ar).

Table S1: Product distribution and associated methane conversion for Pd-iC-CeO_2

Temperature (°C)	Product Yield (μmol)			Oxygenate Selectivity (%)	CH_4 Conversion (%)
	CH_3OH	HCOOH	CO_2		
75	2.9	0	0	100	0.1
90	1.6	0	3.4	32	0.2
100	3.2	0.4	2.7	57	0.3
110	4.7	4.1	4.5	66	0.5
125	0.4	0	7.7	6	0.3

Reaction Conditions: 25 mg catalyst, 20 atm total pressure (20% CH_4, balance Ar), 2.46 mmol initial methane charge, 15 mL 0.5 M H_2O_2 (aq), 800 rpm mixing.

Table S2. Methane to methanol comparison to state-of-the-art catalytic materials with Pd-iC-CeO_2

**Catalyst**	**Loading**	**Reaction Media**	**CH_4 (bar)**	**Temp. (°C)**	**Oxy. Yield (μmol/g_{cat})**	**Oxy. Selectivity (%)**	**Ref**
AuPd/TiO_2	2.5wt%Au 2.5wt%Pd	0.5 M H_2O_2	30	50	1458	90	1
Rh/ZrO_2	0.3wt%Rh	0.5 M H_2O_2	28.5	70	52.47	75	2
IrCuPd/ZSM5	0.5wt%Rh	O_2+CO	20	150	1490	85	3
CuFAU	9.3wt%Cu	O_2 (g)	15	360	360	93	4
AuPd/ZSM-5	3.2wt%Au 1.7wt%Pd	*in situ* H_2O_2	0.48	70	4731	92	5
Pd_1/TiO_2	0.2wt%Pd	H_2O_2 hν (photo)	20	25	180	94	6
**Pd-iC-CeO_2**	**4wt%Pd**	**0.5 M H_2O_2**	**4**	**75**	**112**	**100**	**This Work**
**Pd-iC-CeO_2**	**4wt%Pd**	**0.5 M H_2O_2**	**4**	**110**	**350**	**65**	**This Work**

1. Ab Rahim, M. H.; Armstrong, R. D.; Hammond, C.; Dimitratos, N.; Freakley, S. J.; Forde, M. M.; Morgan, D. J.; Lalev, G.; Jenkins, R. L.; Lopez-Sanchez, J. A.; et al. Low temperature selective oxidation of methane to methanol using titania supported gold palladium copper catalysts. *Catal. Sci. Technol.* **2016**, *6* (10), 3410-3418.
2. Kwon, Y.; Kim, T. Y.; Kwon, G.; Yi, J.; Lee, H. Selective Activation of Methane on Single-Atom Catalyst of Rhodium Dispersed on Zirconia for Direct Conversion. *Journal of the American Chemical Society* **2017**, *139* (48), 17694-17699.
3. Li, M.; Shan, J.; Giannakakis, G.; Ouyang, M.; Cao, S.; Lee, S.; Allard, L. F.; Flytzani-Stephanopoulos, M. Single-step Selective Oxidation of Methane to Methanol in the Aqueous Phase on Iridium-based Catalysts. *Appl. Catal., B* **2021**, 120124
4. Sushkevich, V. L.; van Bokhoven, J. A. Methane-to-Methanol: Activity Descriptors in Copper-Exchanged Zeolites for the Rational Design of Materials. *ACS Catal.* **2019**, *9* (7), 6293-6304
5. Jin, Z.; Wang, L.; Zuidema, E.; Mondal, K.; Zhang, M.; Zhang, J.; Wang, C.; Meng, X.; Yang, H.; Mesters, C. Hydrophobic zeolite modification for in situ peroxide formation in methane oxidation to methanol. *Science* **2020**, *367* (6474), 193-197
6. Ab Rahim, M. H.; Armstrong, R. D.; Hammond, C.; Dimitratos, N.; Freakley, S. J.; Forde, M. M.; Morgan, D. J.; Lalev, G.; Jenkins, R. L.; Lopez-Sanchez, J. A.; et al. Low temperature selective oxidation of methane to methanol using titania supported gold palladium copper catalysts. *Catal. Sci. Technol.* **2016**, *6* (10), 3410-3418.

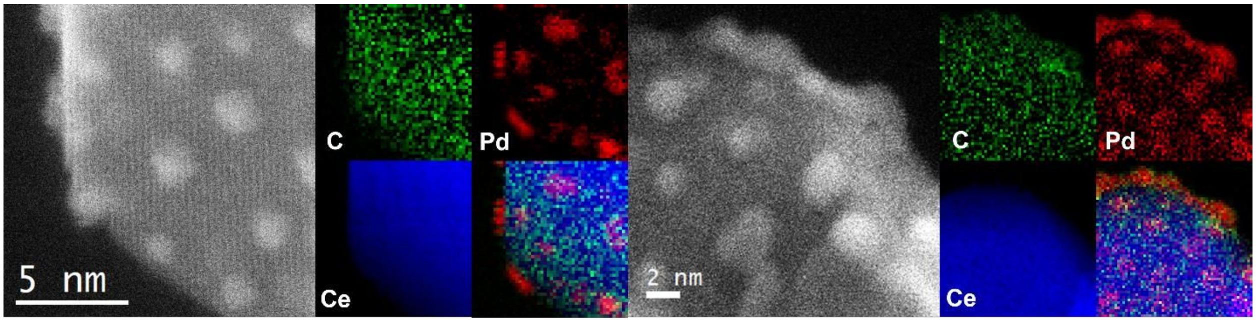

Figure S2: STEM-EELS ex situ imaging of Pd-iC-CeO_2 for the (left) fresh catalyst (right) after 1 hr reaction condition. Reaction Conditions: 25 mg catalyst, 0.5 M H_2O_2 (aq), 800 rpm stir rate, 75 °C, 20 bar initial pressure.

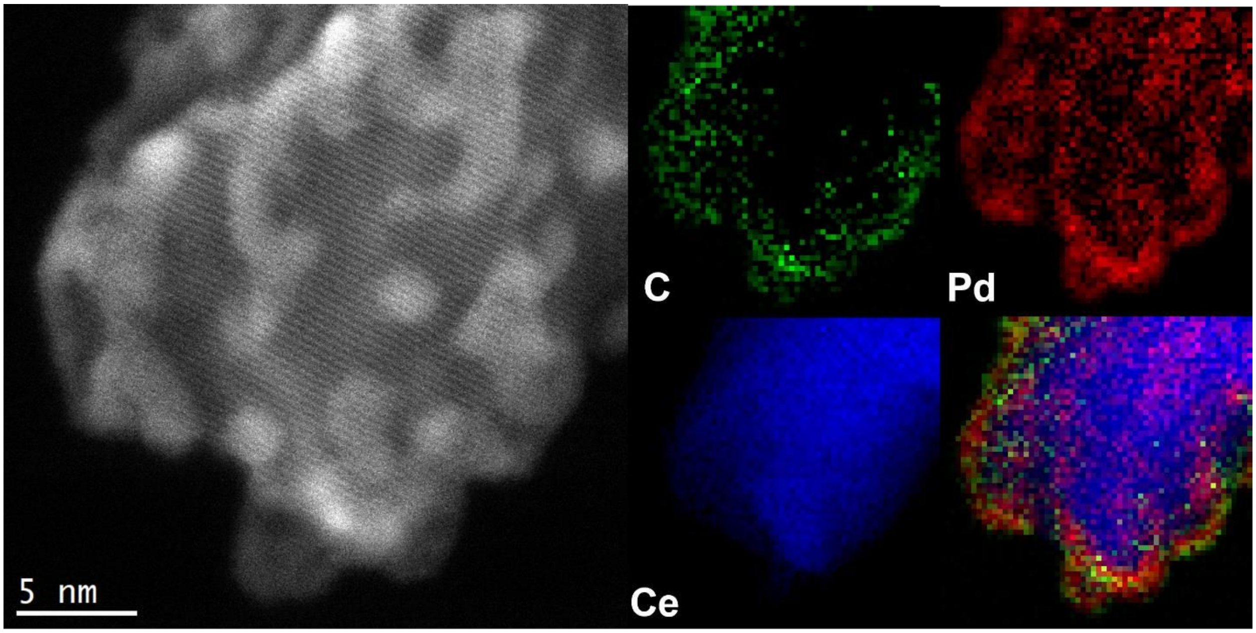

Figure S3: STEM EELS for Pd-iC-CeO_2 after 3 hr of reaction time Reaction conditions: 25 mg catalyst, 0.5 M H_2O_2 (aq), 800 rpm stir rate, 75 °C, 20 bar initial pressure.

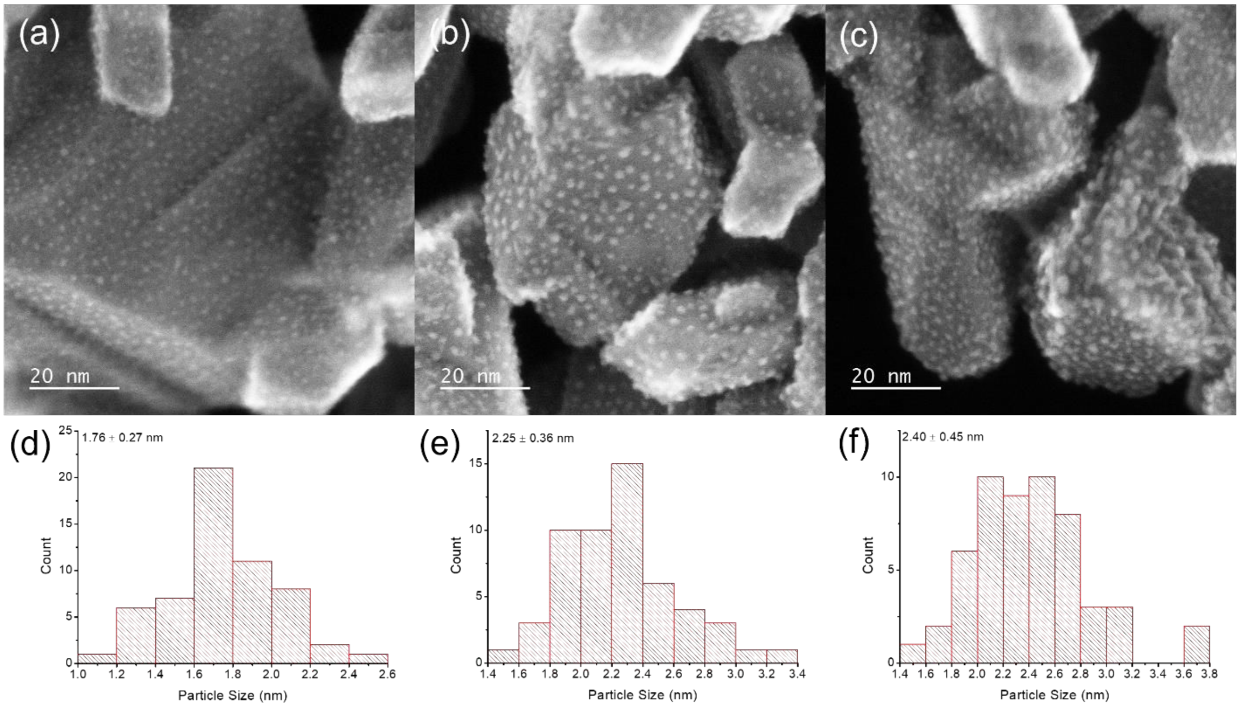

Figure S4: STEM imaging of Pd-iC-CeO_2 for (a) fresh as synthesized (b) after 1 hr of reaction time (c) after 3 hr of reaction time. Particle size distribution for (d) fresh sample, (e) 1 hr reaction time and (f) 3 hr of reaction time.

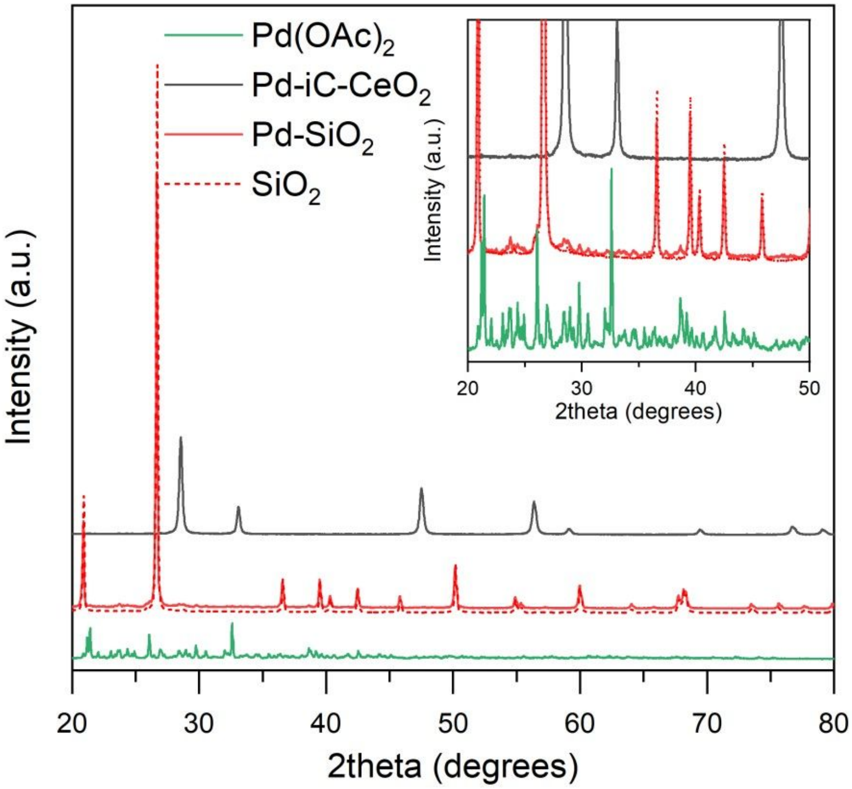

Figure S5: Diffraction patterns for ball milled palladium supported on ceria (Pd-iC-CeO_2), silica (Pd-SiO_2), bare silica, and $Pd(OAc)_2$ salt. Diffraction patterns of the precursor acetate salt can be observed on Pd-SiO_2, while no additional Pd or $Pd(OAc)_2$ peaks are detected on Pd-iC-CeO_2.

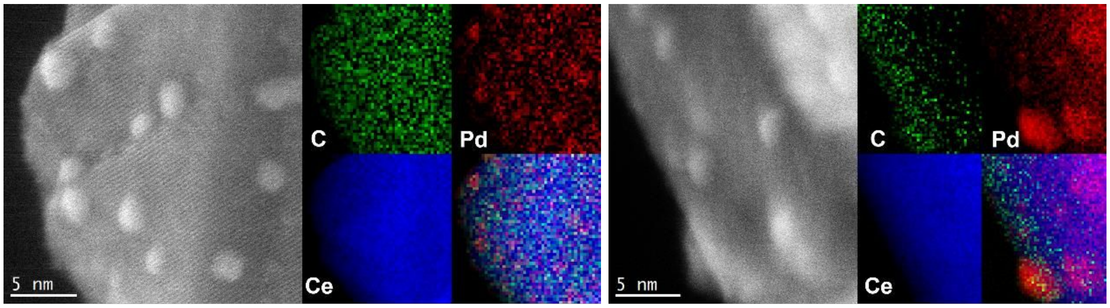

Figure S6: STEM-EELS ex situ imaging of Pd-iC-CeO_2 post oxidation treatment in air for 1 hr at 500 °C (left) post oxidative treatment (right) after1 hr reaction condition. Reaction Conditions: 25 mg oxidized Pd-iC-CeO_2, 0.5 M H_2O_2 (aq), 800 rpm stir rate, 75 °C, 20 bar initial pressure.

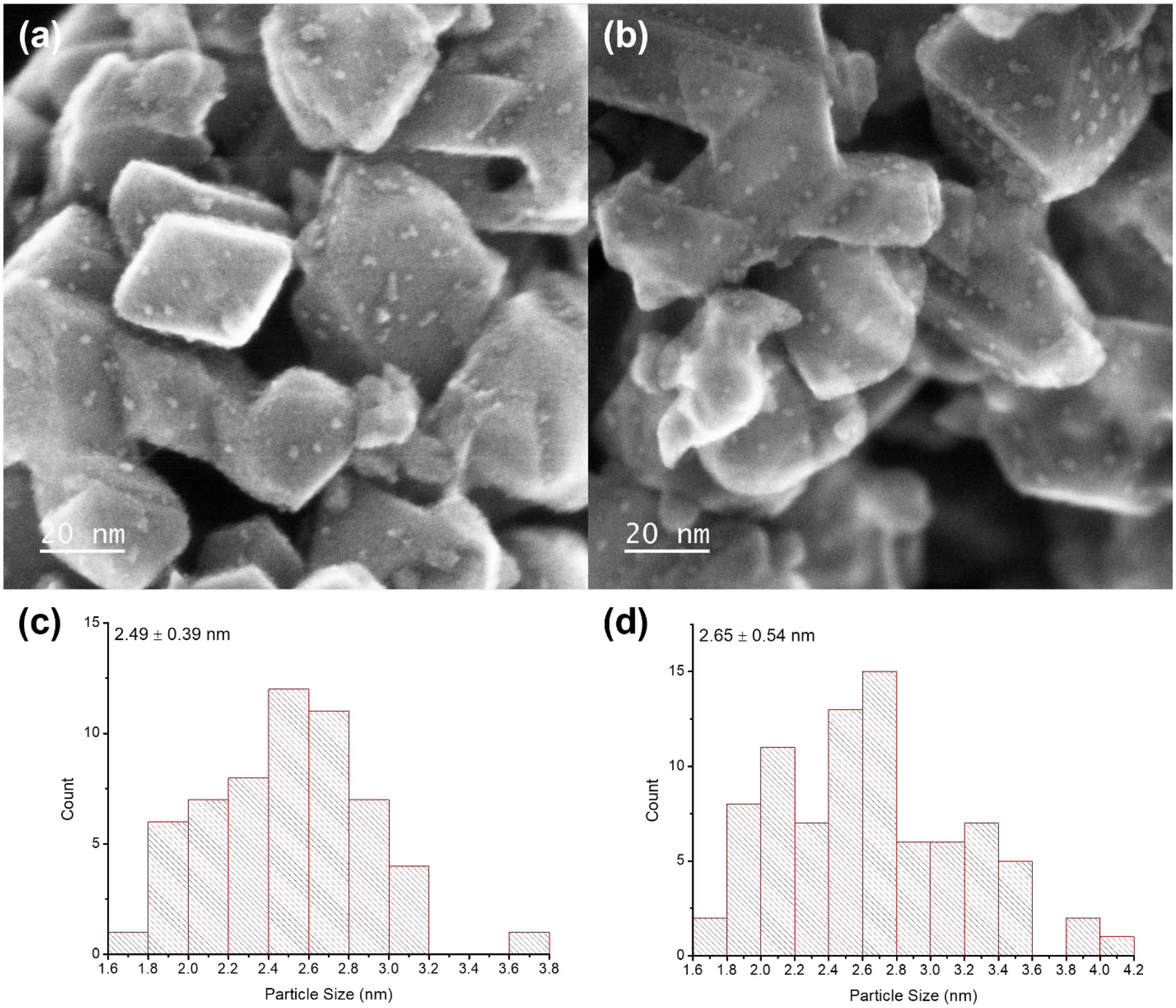

Figure S7: STEM particle sizing for Pd-iC-CeO_2 after (a) and oxidative treatment and (b) oxidative pretreatment and 1 hr of MtM reaction time. Particle size distribution for after (c) oxidative treatment and (d) oxidative treatment and 1 hr of MtM reaction time.

Dispersion Calculation

Assume Pd^0 crystallites have an FCC structure and are semi spherical as shown in the STEM images, with the upper half exposed, the dispersion is as follows:

$$D = \frac{N_S}{N_T} = 6\,\frac{V_m/a_m}{d_{VA}}$$

Where,

N_S is the number of palladium atoms present on the surface

N_t is the total number of palladium atoms in the catalyst

V_m is the volume occupied by one palladium in the bulk

a_m is the area occupied by one palladium atom on the polycrystalline surface

d_{VA} is the mean particle diameter size of the metallic palladium via STEM

Dispersion of Pd-iC-CeO_2, with an average particle size of 1.7 nm for the fresh sample and 2.4 nm for the spent 3 hr samples is **22%** and **16%**, respectively.

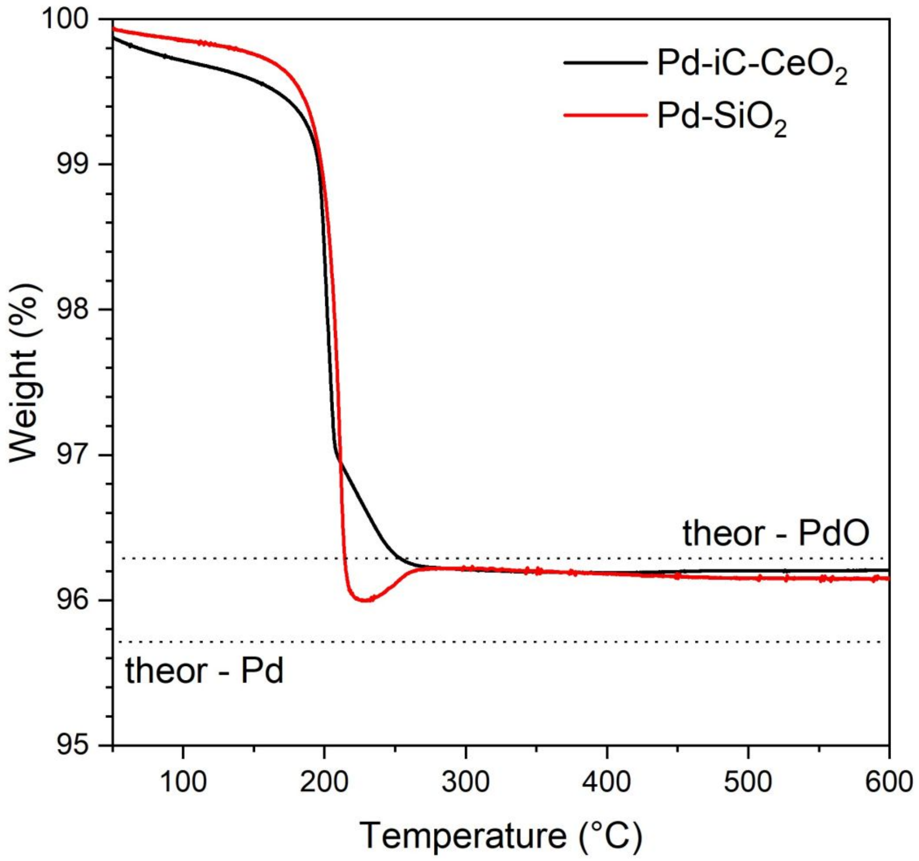

Figure S8: Thermogravimetric Analysis (TGA) of as synthesized Pd-iC-CeO_2 and Pd-SiO_2. The decomposition of the Palladium acetate salt can be observed from the sudden weight loss at 200°C, which corresponds to a total release of the acetate ligands and the oxidation of supported Pd into PdO.

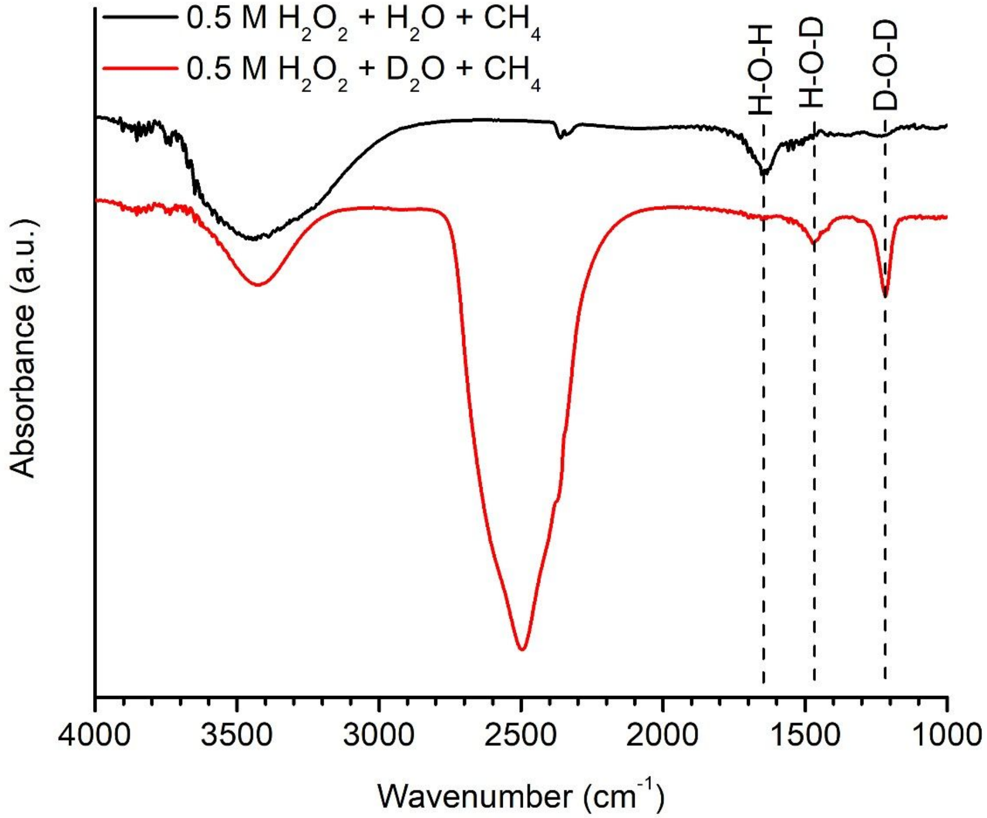

Figure S9: Full range in situ MtM ATR for Pd-iC-CeO_2 using either 0.5 M H_2O_2 in deuterium or DI water.

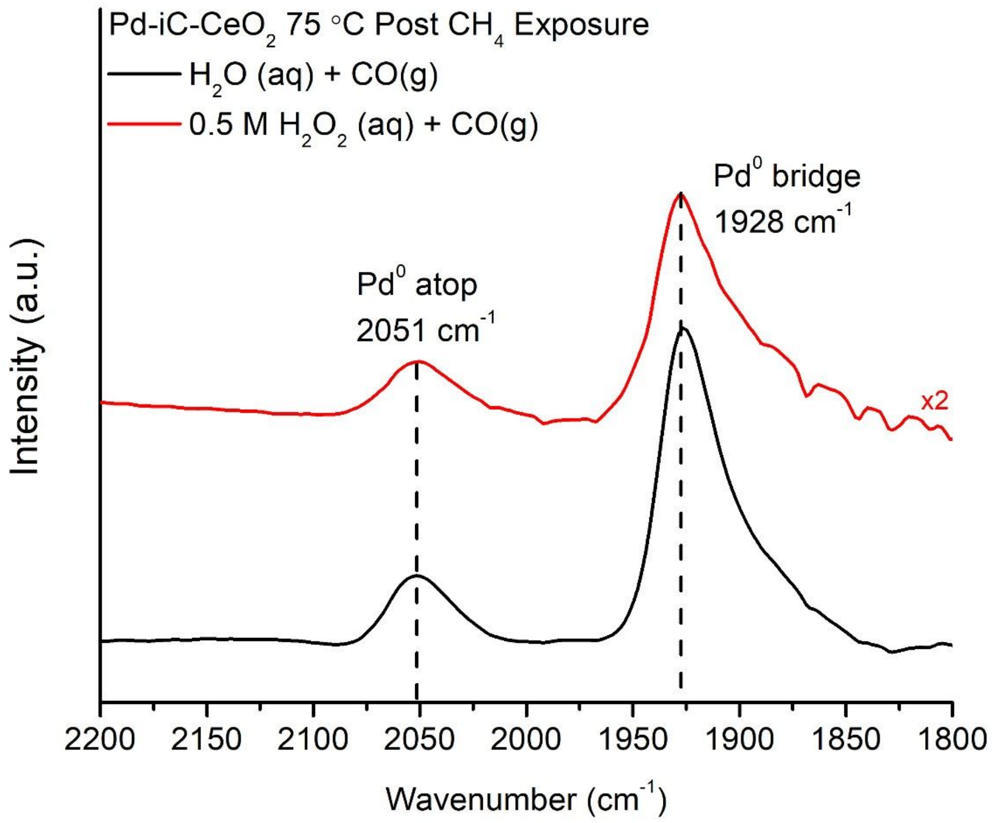

Figure S10: in situ CO-ATR at 75 °C post methane exposure in either solvent alone (H_2O + CO) or full reaction conditions (0.5 M H_2O_2 + H_2O + CO).

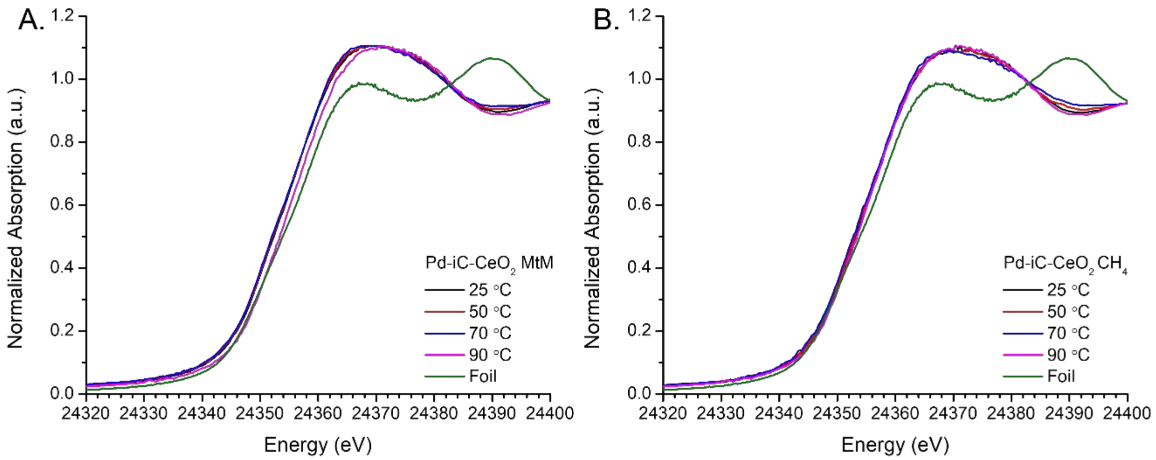

Figure S11: in situ Pd K edge XANES for Pd-iC-CeO_2 at 34 bar total pressure under (A) 20 bar partial pressure of methane and 0.1 M H_2O_2 (aq) flow and (B) 20 bar partial pressure of methane and DI water. All flows were set to 0.01 mL/min and data was collected via a PIPS detector.

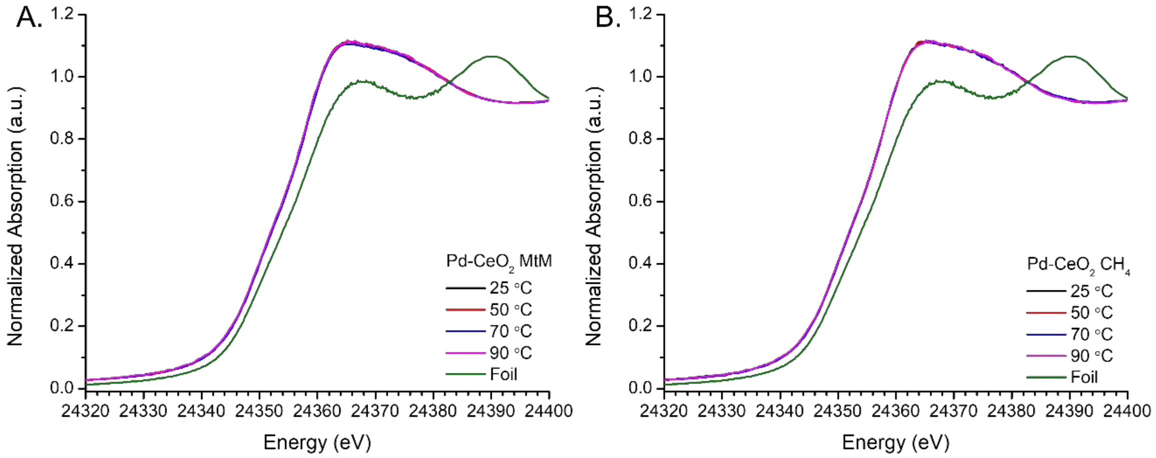

Figure S12: in situ Pd K edge XANES for Pd -CeO_2 at 34 bar total pressure under (A) 20 bar partial pressure of methane and 0.1 M H_2O_2 (aq) flow and (B) 20 bar partial pressure of methane and DI water. All flows were set to 0.01 mL/min and data was collected via a PIPS detector.

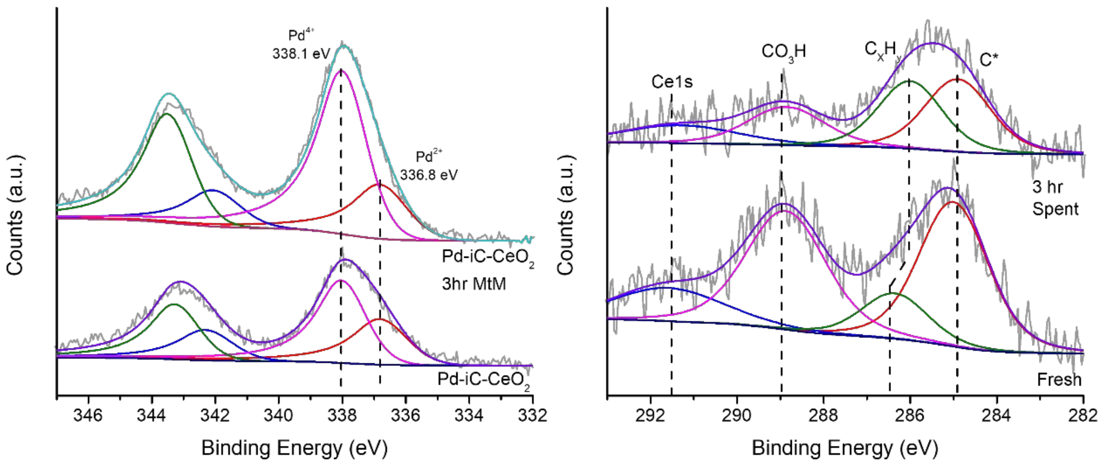

Figure S13: XPS spectra of as synthesized Pd-iC-CeO_2 and after 3 hr reaction time (left) Pd3d and (right) C1s.

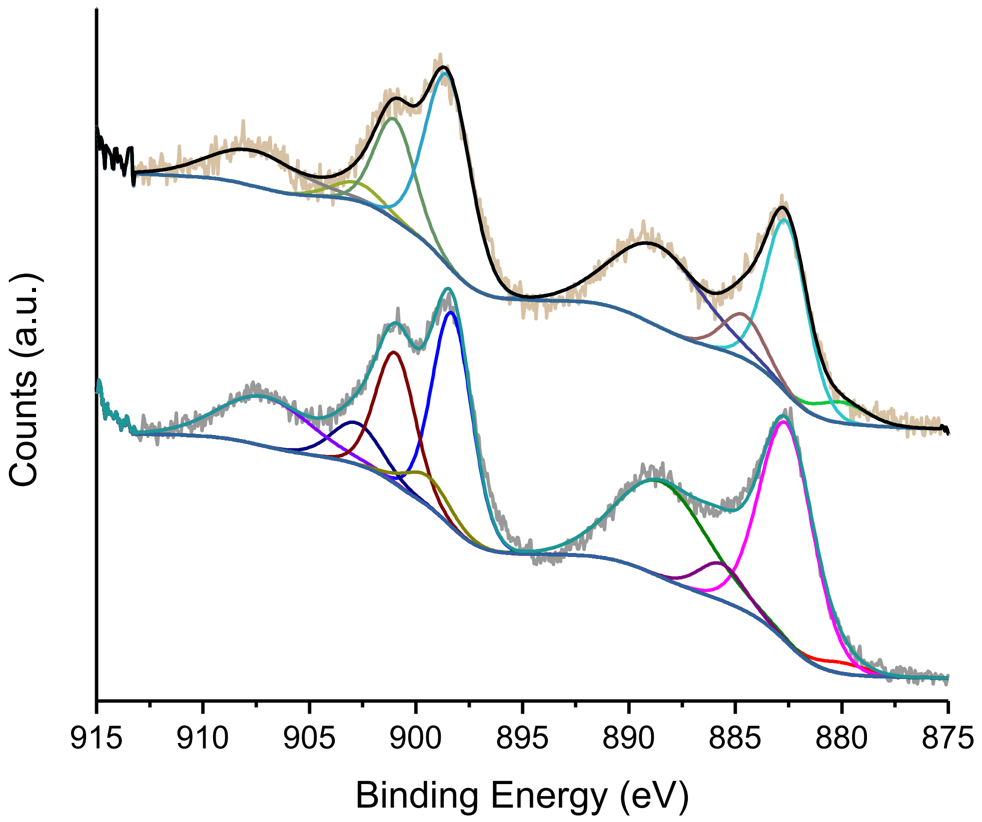

Figure S14: Ce3d ex situ XPS spectra of Pd-iC-CeO_2 (bottom) as synthesized and (top) after MtM reaction for 3 hr.

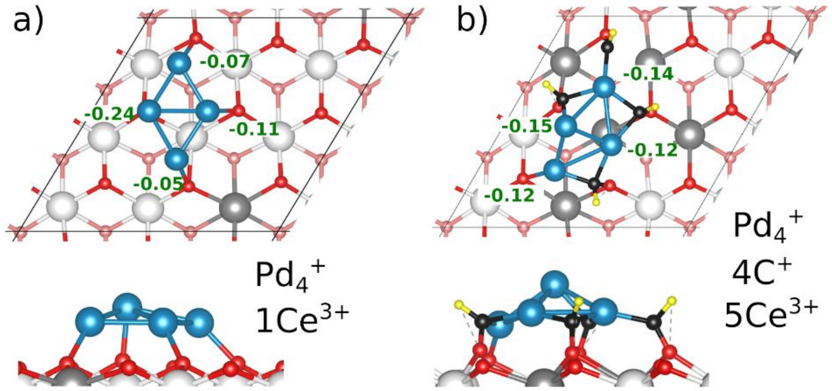

Figure S15: a) Pd-CeO_2 and b) Pd-iC-CeO_2 theoretical models used in this study. Color coding: Pd atoms are light blue, Ce^{4+} white, Ce^{3+} grey, O atoms of the first layer red, while those of the second layer are light red, C atoms black and H atoms yellow. The Bader charge donated by each Pd atom, relative to the Pd atom in the gas phase, is indicated in green.

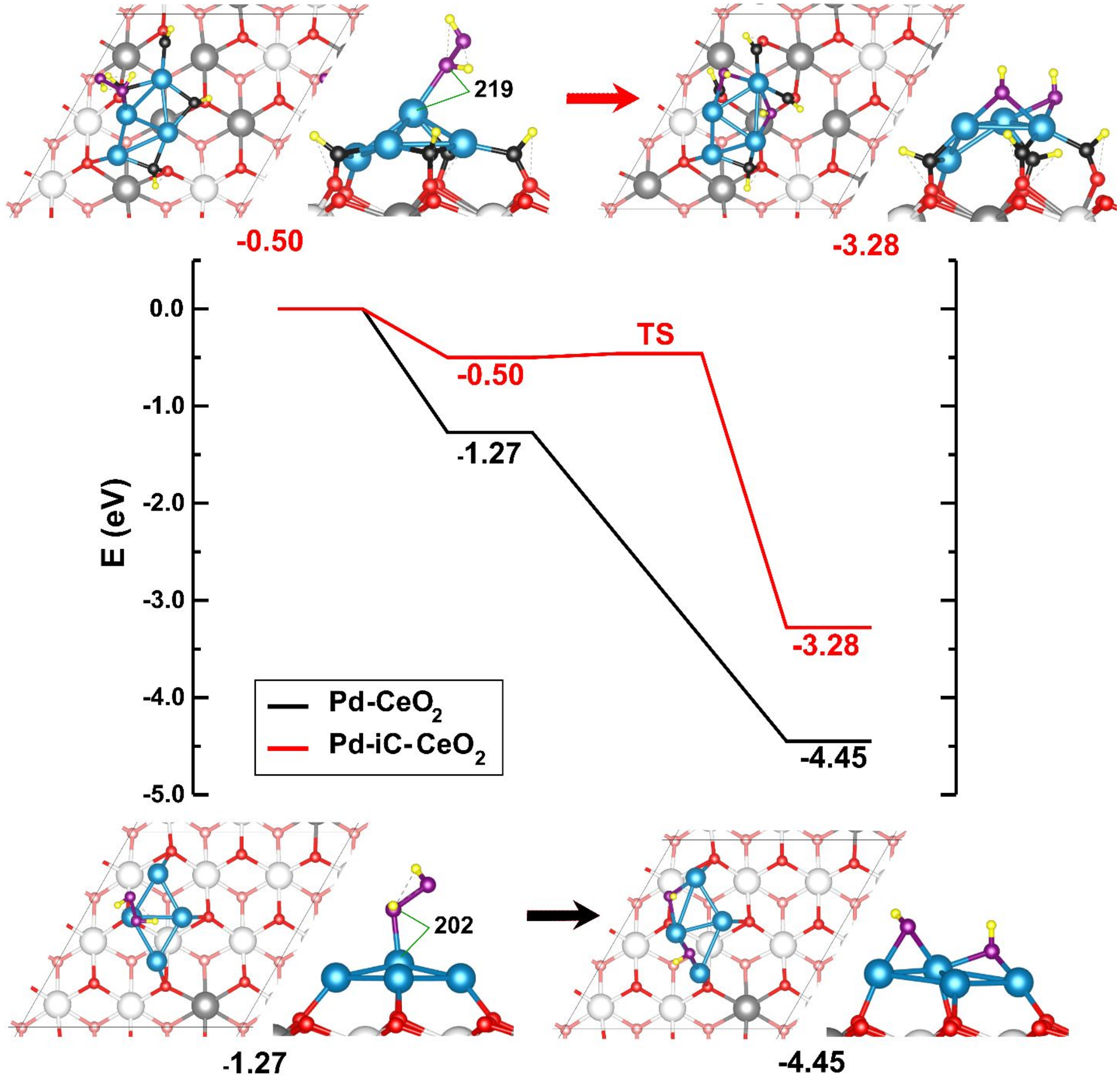

Figure S16: Activation of H_2O_2 on Pd-CeO_2 (black path) and Pd-iC-CeO_2 (red path). In the case of the molecular adsorbed state the H–Pd distance is indicated in pm. The adsorption energy of each stable state and the activation energy associated with the transition states relative to their precursor states are included.

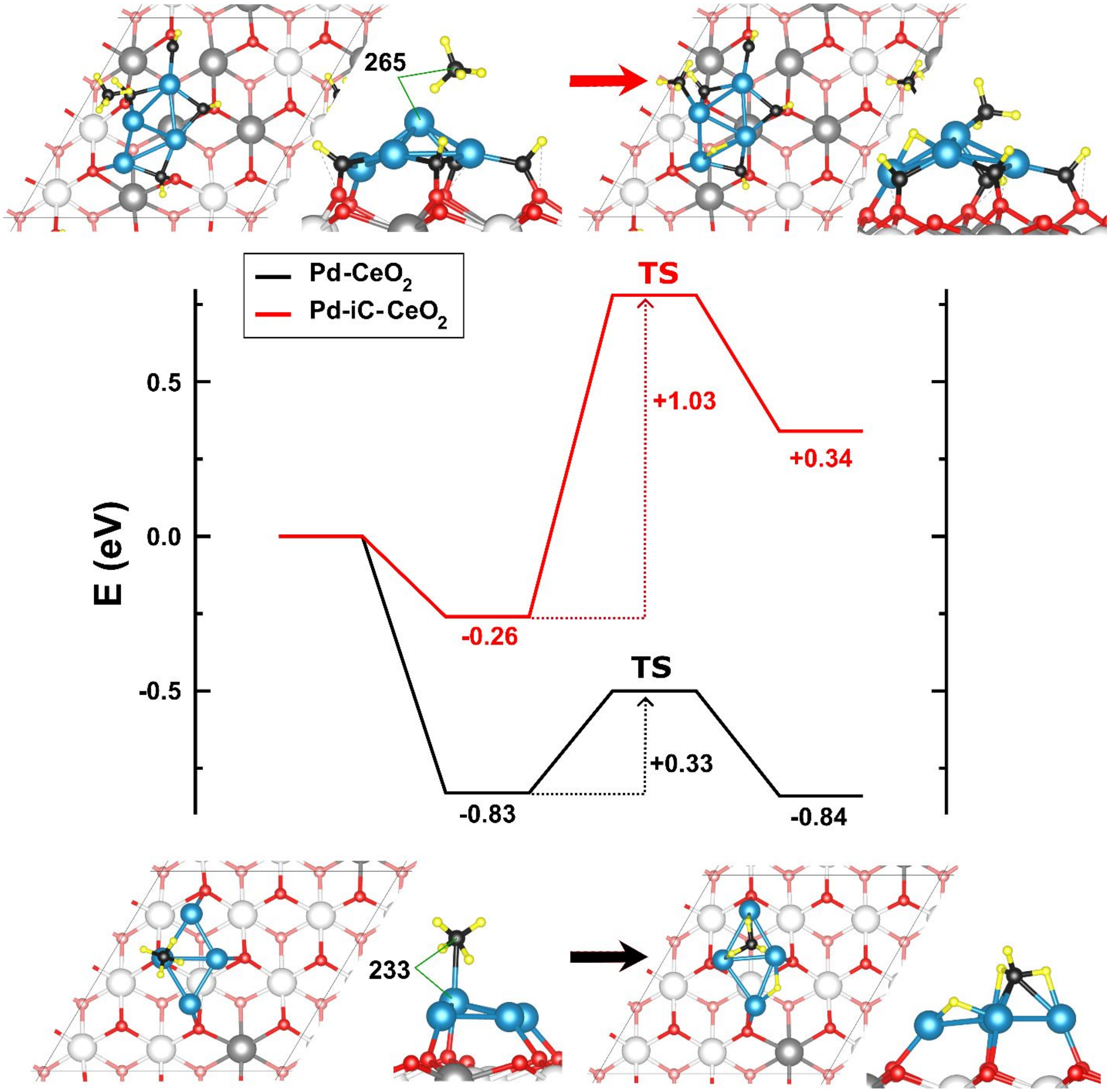

Figure S17: Activation of CH_4 on Pd-CeO_2 (black path) and Pd-iC-CeO_2 (red path). In the case of the molecular adsorbed state the C–Pd distance is indicated in pm. The adsorption energy of each stable state and the activation energy associated with the transition states relative to their precursor states are included.

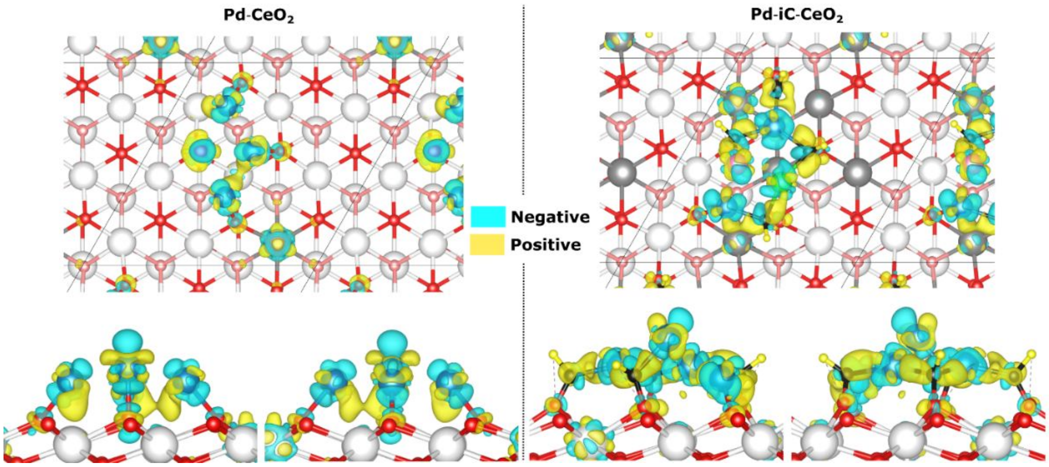

Figure S18: Top (upper) and side (bottom) views of the charge density difference plots (CDDP) for two model catalysts: Pd-CeO$_2$ and Pd-iC-CeO$_2$. The CDDP is computed as follows: ρ(Pd-CeO$_2$/Pd-iC-CeO$_2$) - ρ(CeO$_2$/iC-CeO$_2$) - ρ(Pdgas). Positive/negative differences (Δρ) are visually represented using yellow/cyan colors.

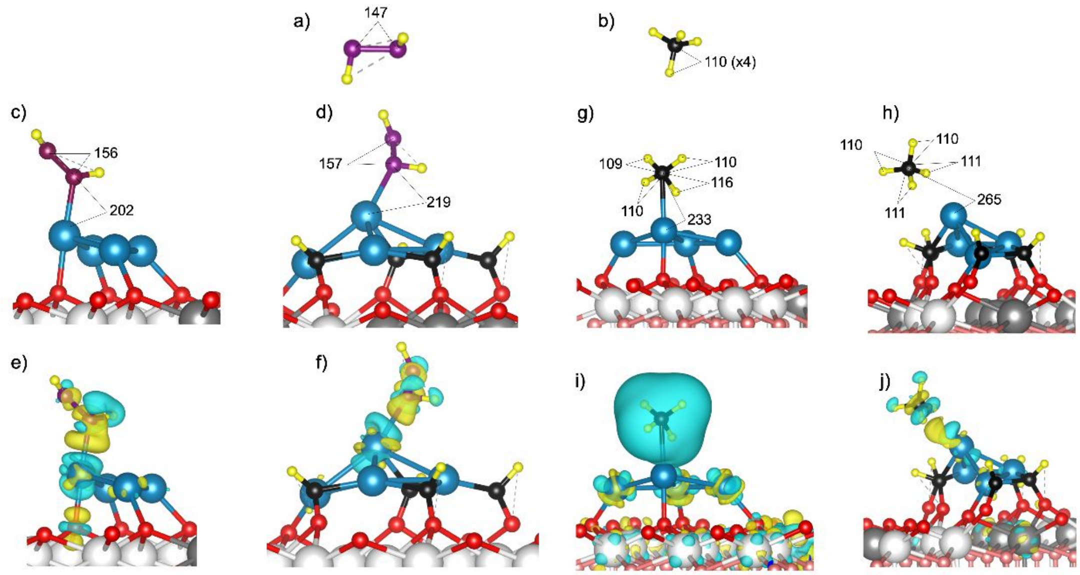

Figure S19: (a) and (b) show the isolated structures of H_2O_2 and CH_4, respectively, with bond lengths in picometers. (c) and (d) display the adsorption geometries of H_2O_2 on Pd-CeO$_2$ and Pd-iC-CeO$_2$, respectively, indicating bond lengths and changes upon adsorption. (e) and (f) illustrate the charge density difference plots (CDDP) upon H_2O_2 adsorption on Pd-CeO$_2$ and Pd-iC-CeO$_2$, respectively. (g) and (h) show the adsorption geometries of CH_4 on Pd-CeO$_2$ and Pd-iC-CeO$_2$, respectively, indicating bond lengths and changes upon adsorption. (i) and (j) illustrate the CDDP upon CH_4 adsorption on Pd-CeO$_2$ and Pd-iC-CeO$_2$, respectively. The CDDP is computed as follows: ρ(H_2O_2/CH_4/Catal.) - ρ(Catal.) - ρ(H_2O_2gas/CH_4gas). Positive/negative differences (Δρ) are visually represented using yellow/cyan colors.

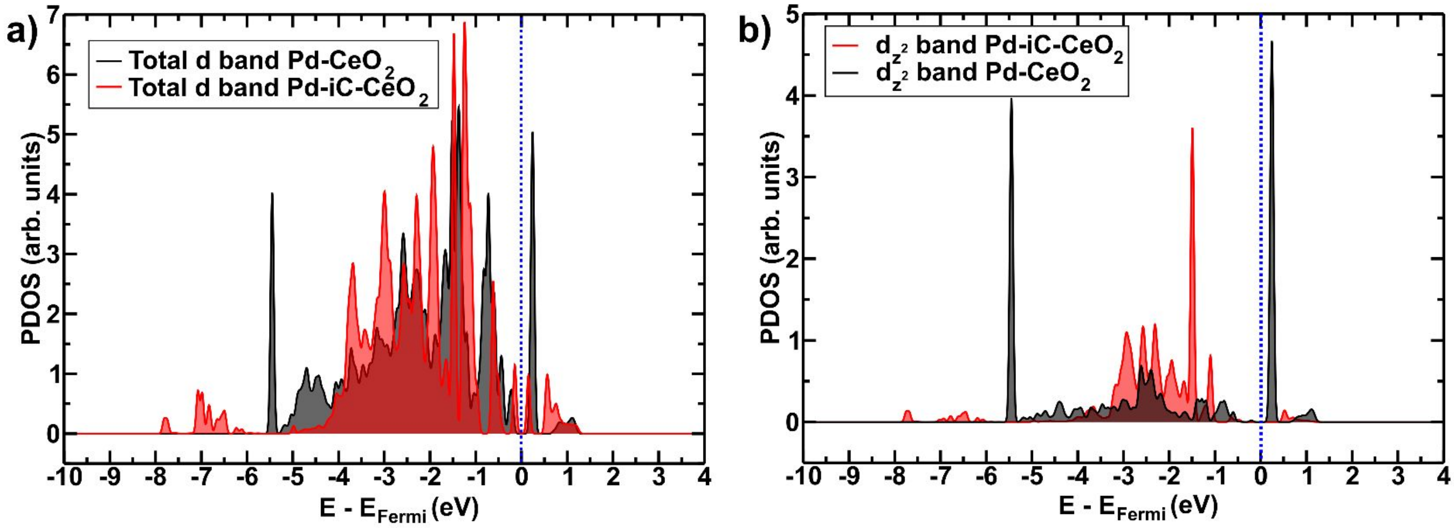

Figure S20: a) d-projected density of states (PDOS) and b) d_{z^2}-projected DOS on the Pd atom over which CH_4 and H_2O_2 dissociate on Pd-CeO_2 and Pd-iC-CeO_2 (Fig. S17 and S18). The PDOS are referenced to the Fermi energy which is indicated in the blue dotted line.

Mechanism of methanol formation on Pd-CeO_2

Figure S21 illustrates the mechanism found for methanol formation on the Pd-CeO_2 model catalyst, and Figure S22 shows images corresponding to each reported stable state.

As H_2O_2 adsorbs more strongly than CH_4 and activates without barrier, we have started with (a), the adsorption of H_2O_2 with an energy of –1.27 eV and (b), followed by the activation of the H_2O_2 molecule, resulting in the final state of 2OH adsorbed (2OH*) adsorbed on Pd with energy of –4.45 eV (see Figure 20b).

Then, methane was adsorbed in the presence of the 2OH* with an energy of –0.18 eV (step (c) in Figure S21 and state (c) in Figure S22). It is notable that the CH_4 adsorption energy decreases by 0.65 eV (0.83 – 0.18 eV) compared to the hydroxyl-free case. The final state: (d) CH_3*+H*+2OH* where all species are dissociatively adsorbed on Pd, has an energy of –4.10 eV. This step is endothermic by 0.53 eV (–4.10 – (–4.63) eV). Calculation of the activation barrier (E_{act}) showed no transition state (TS), thus the activation energy of (c) CH_4*+2OH* to (d) CH_3*+H*+2OH* equals the reaction energy of 0.53 eV. This value is by 0.2 eV larger compared to the case of the surface without a dissociatively pre-adsorbed H_2O_2 molecule (cf. Figure S17).

At this point we already have the necessary ingredients (CH_3* and OH*) to form CH_3OH. Then, we evaluated the path from (d) CH_3*+H*+2OH* to (d') CH_3OH*+H*+OH (red dotted path Figure S21) and found an activation barrier of E_{act}=1.53 eV. In addition to this high activation barrier the process is endothermic with a reaction energy of E_{react}=+0.16 eV, which indicates that even if CH_3OH were formed it would tend to dissociate into CH_3*+OH* since the reverse activation barrier is lower (1.37 eV). Since this path is not possible, a second H_2O_2 molecule was adsorbed and activated between the (e) CH_3*+H*+2OH*+H_2O_2* and (f) CH_3*+H*+2OH*+OH_{int}*+H_2O* states. Note that the second H_2O_2 does not dissociate into 2OH*, but forms with the H* a H_2O molecule, leaving also an OH adsorbed at the PdCeO_2 interface (OHint). This process has an activation barrier of 0.80 eV and is 2.90 eV exothermic. Thus, it is concluded that it is more feasible to adsorb and activate a second H_2O_2 molecule than to form CH_3OH.

Continuing from state (f), the formation of methanol was newly considered, finding a state (f') with an energy of –7.54 eV, so the reaction energy of this step is –0.33 eV but the activation barrier is 1.40 eV. This result is promising because the reaction energy is exothermic, and the activation barrier is lower than that found in methanol formation between steps (d) and (d') of 1.53 eV. Likewise, the adsorption and activation of a third H_2O_2 molecule corresponding to the states (g) 2OH*+CH_3*+H*+OHint*+H_2O*+H_2O_2* and (h) 3OH+CH_3*+H*+2OH_{int}*+H_2O* were analyzed. In this step, H_2O_2* dissociates into OH*+OH_{int}*. As seen in Figure S21, nearly 1 eV is gained by adsorbing H_2O_2*, and its dissociated products produce an exothermic energy of 2.06 eV with an E_{act} = 0.62 eV. Therefore, in this last step, the reaction would continue.

Finally, considering the proposed model, there is no more available space to dissociate a third H_2O_2 molecule without interacting with CH_3*. This aspect will be further explored later with the inclusion of an aqueous environment. Consequently, the formation of CH_3OH was calculated from

state (h), finding a state (i) with an energy of −10.45 eV and an E_{react}= –0.21 eV. The calculation of the reaction path in this step showed an E_{act} = 1.21 eV, the lowest obtained so far for the formation of CH_3OH. Ultimately, the energy required to desorb CH_3OH is 1.14 eV (state (j) in Figure S21).

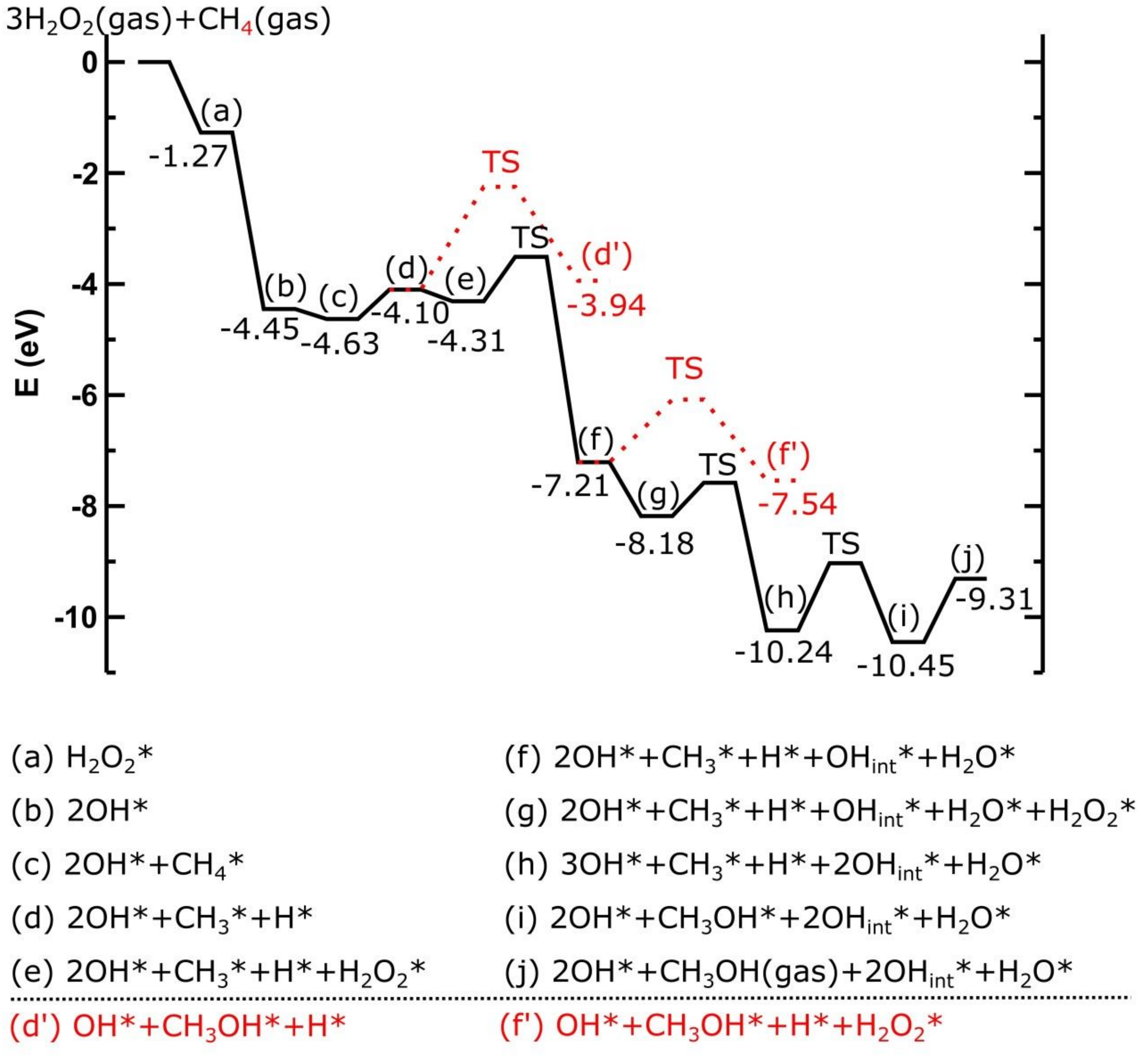

Figure S21: Hydrogen peroxide-assisted methane to methanol formation on Pd-CeO_2. The red dotted lines correspond to evaluations of pathways to methanol formation that were discarded because of their high activation barrier. The adsorption energy of each stable state and the activation energy associated with the transition states relative to their precursor states are included.

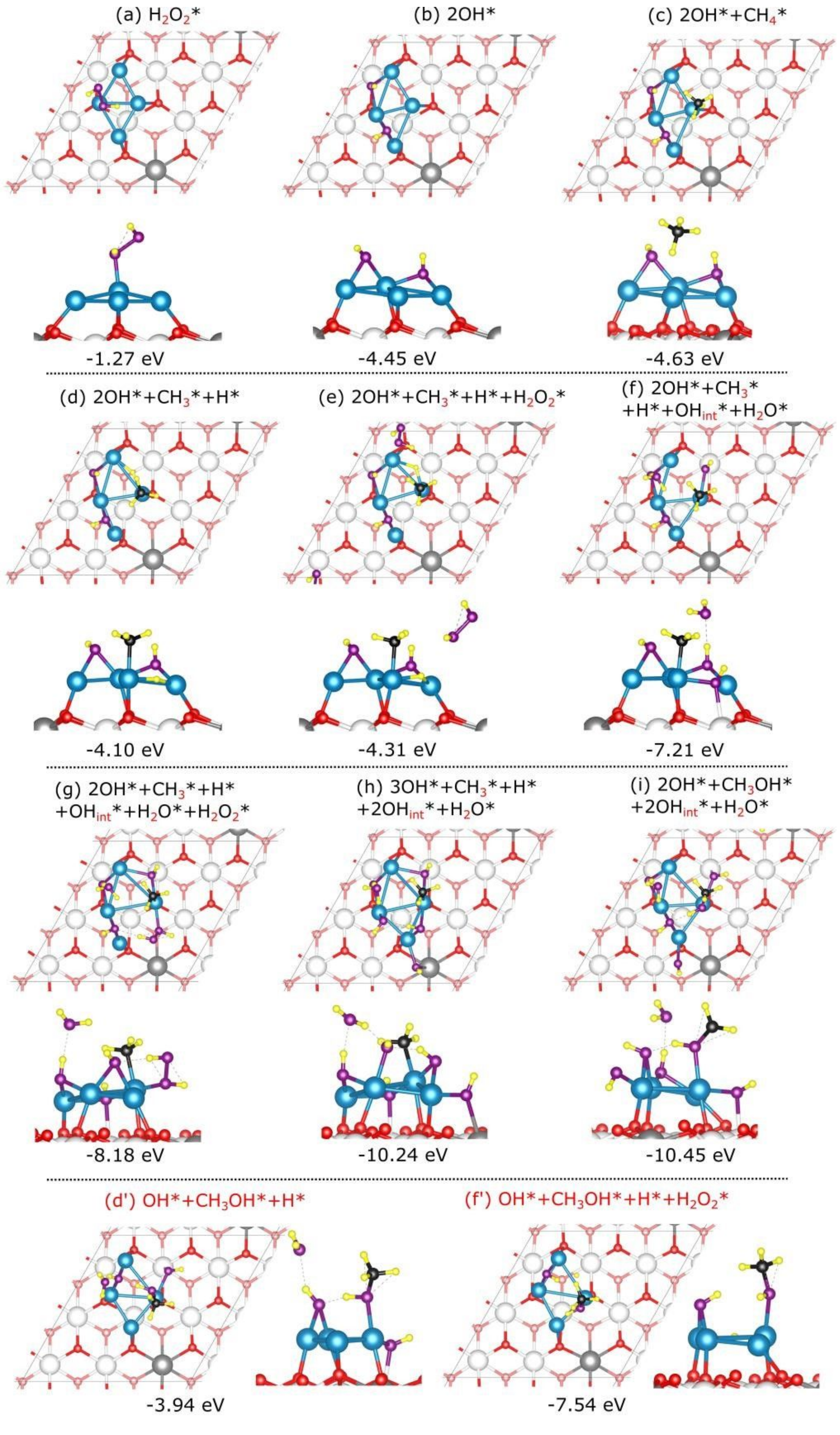

Figure S22: Top and side view of the states belonging to the reaction mechanism in Figure S21.

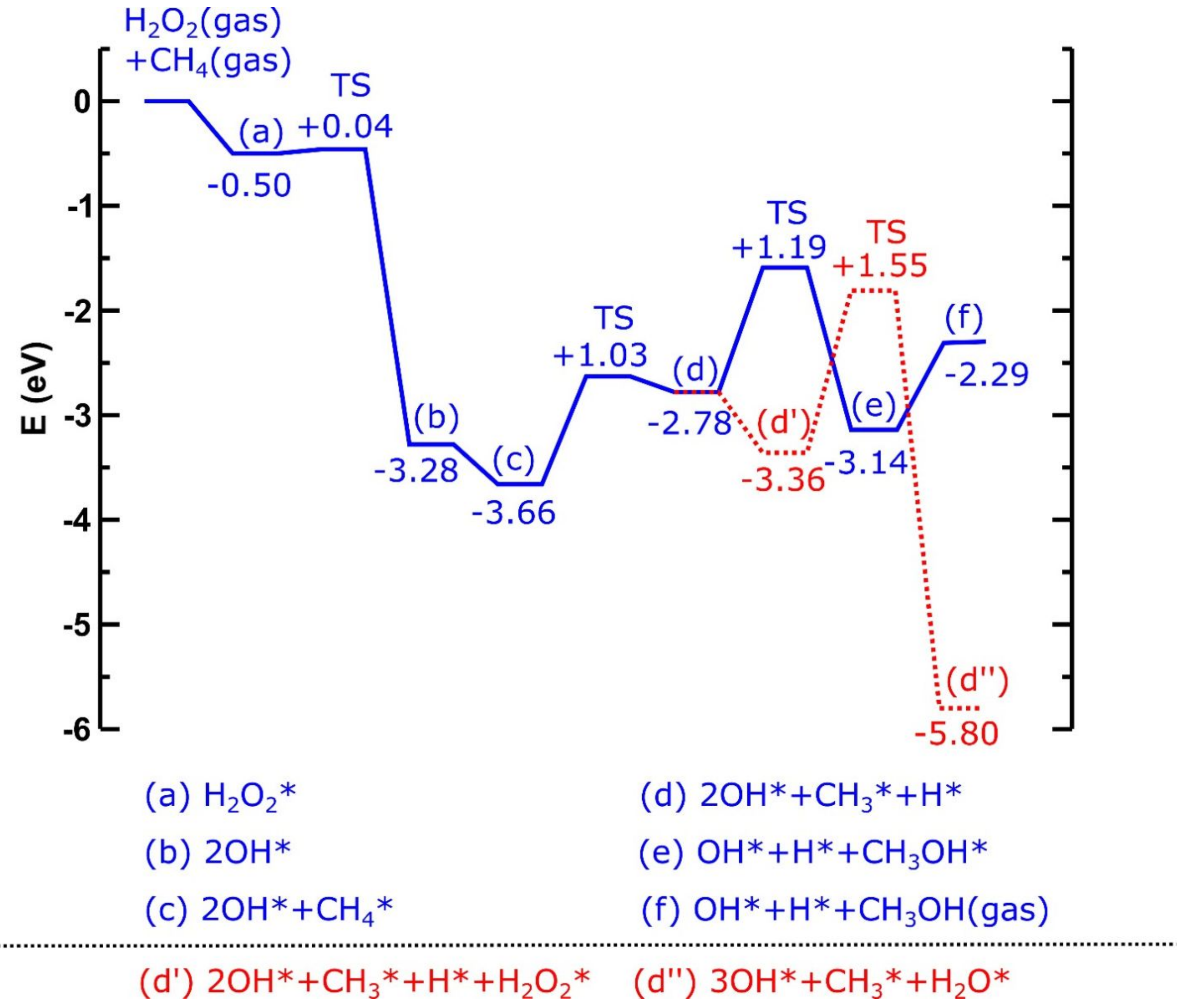

Figure S23: Hydrogen peroxide-assisted methane to methanol formation on Pd-iC-CeO_2. The red dotted lines correspond to evaluations of pathways to H_2O_2 activation that was discarded because of the high activation barrier. The adsorption energy of each stable state and the activation energy associated with the transition states relative to their precursor states are included.

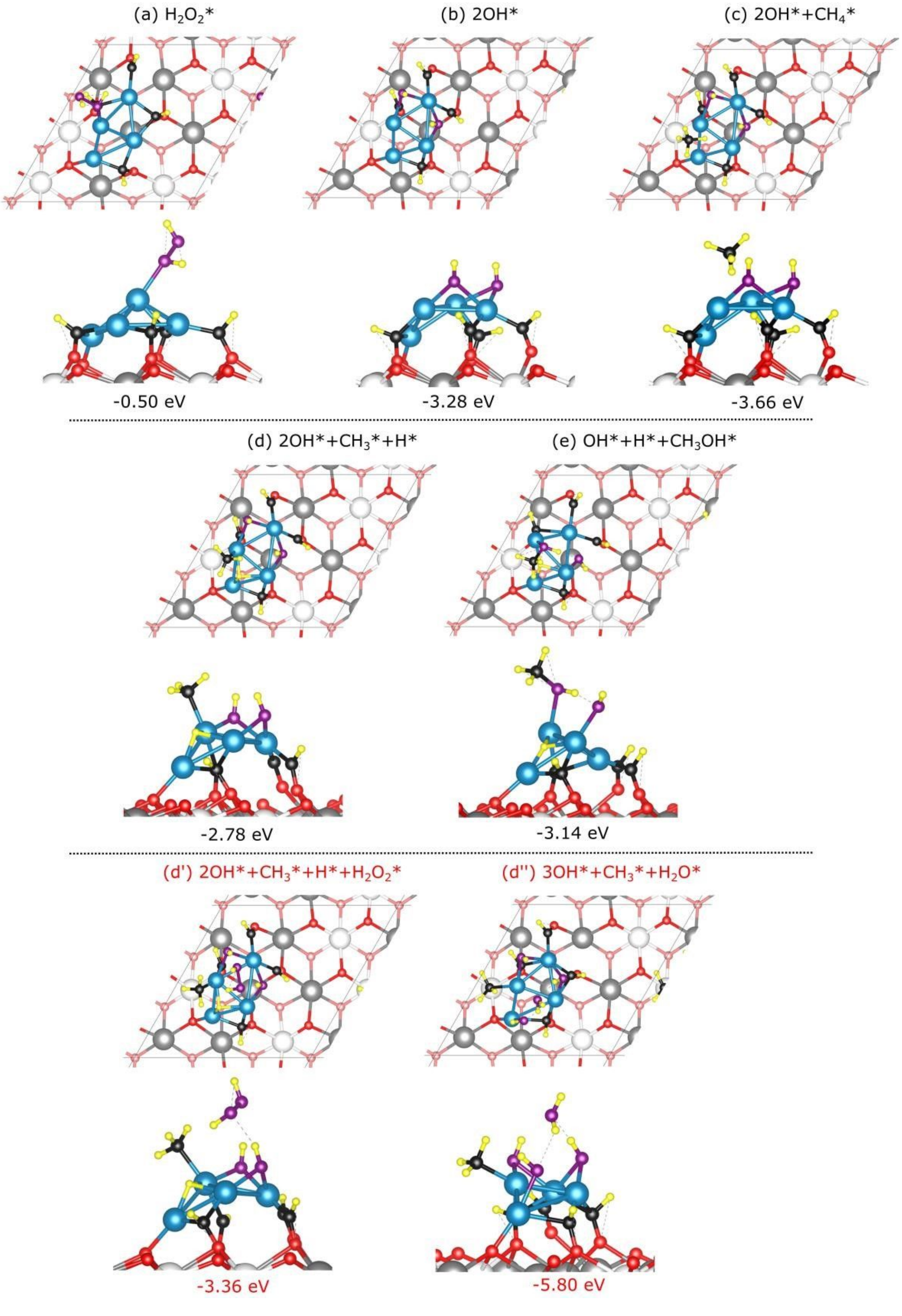

Figure S24: Top and side view of the states belonging to the reaction mechanism in Figure S23.

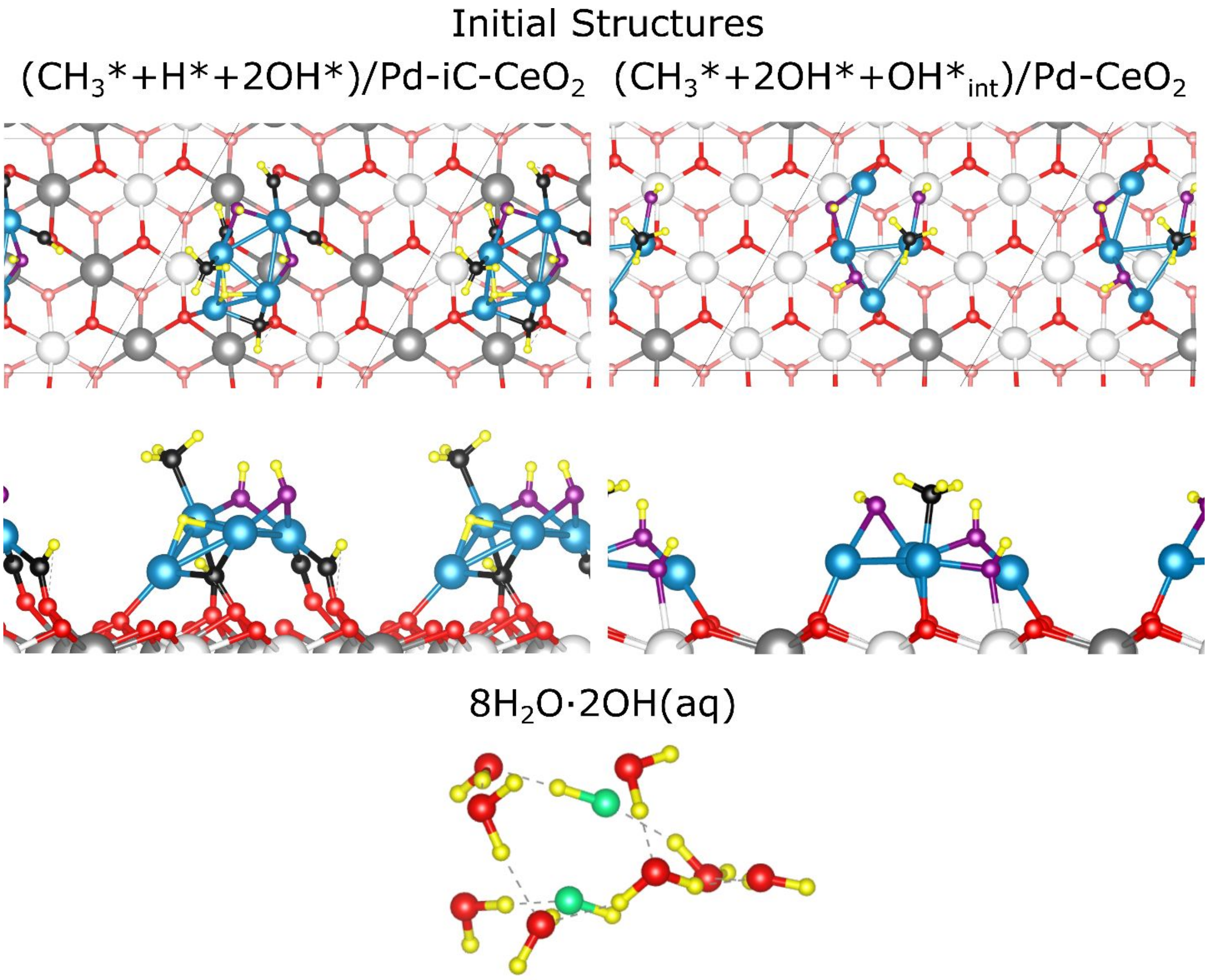

Figure S25: Initial structures for the study of the Eley-Rideal mechanism for CH_3OH formation. Oxygen atoms belonging to the solvated OH are shown in green.

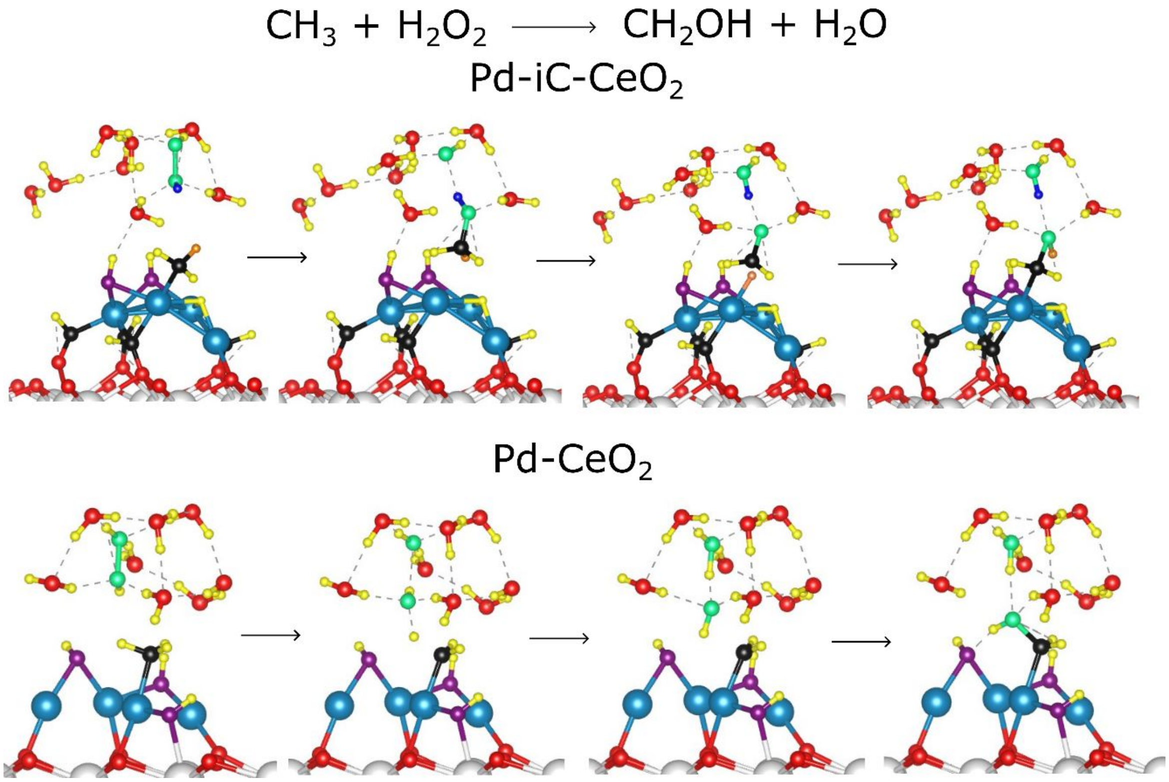

Figure S26: States involved in the formation of CH_2OH over the model catalysts studied.

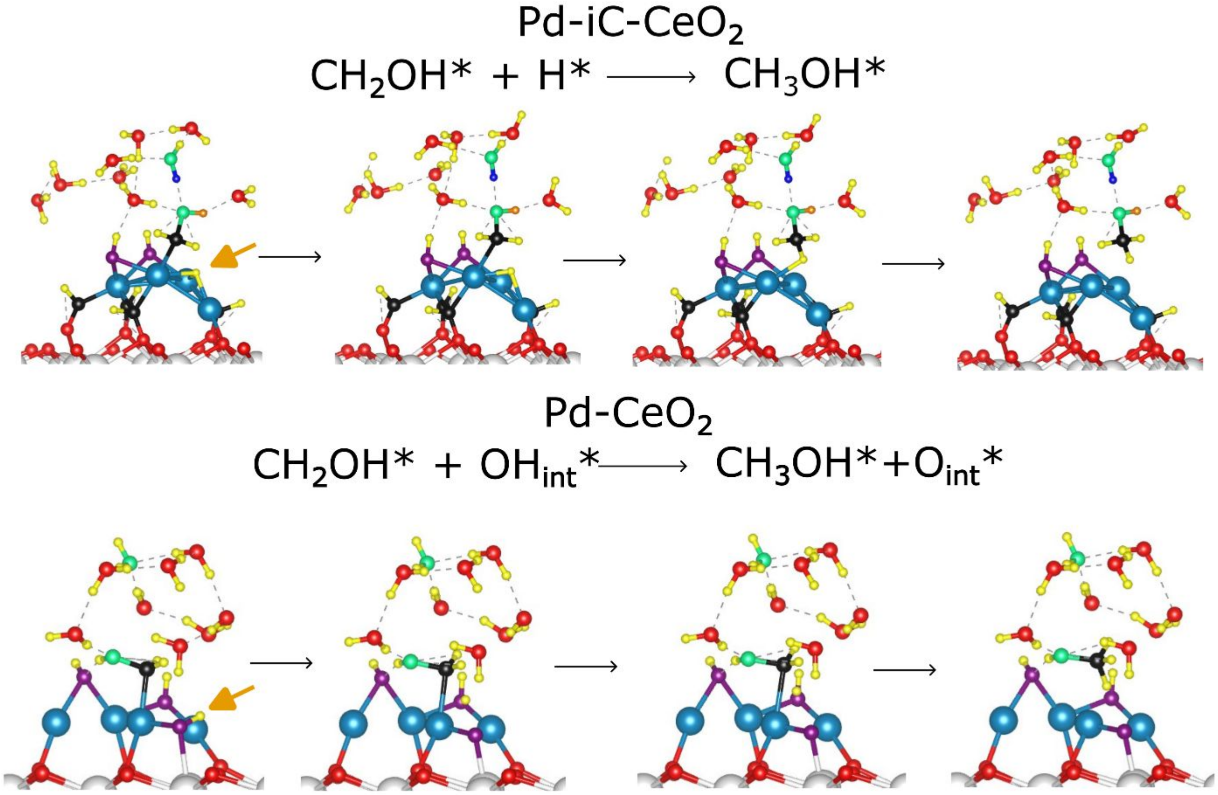

Figure S27: Step for the formation of CH_3OH. The orange arrow at the beginning of both pathways points to the H involved in the formation of methanol.

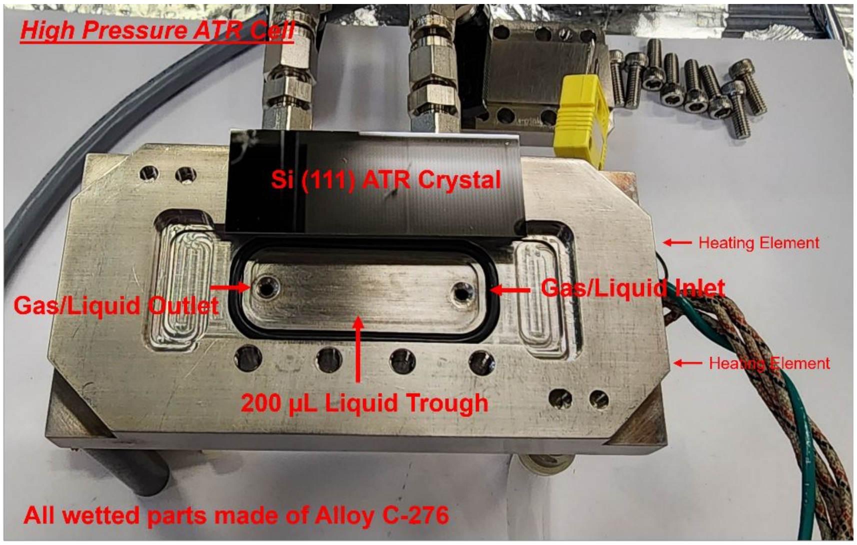

Fig. S28. High pressure commercial ATR-IR cell configuration (Harrick, Horizon Multiple Reflection ATR Unit). Catalyst drop cast onto a 50 x 20 x 2 mm Si (111) ATR crystal using a slurry of catalyst and DI water to mirror solvent conditions. System shown in upside-down configuration for assembly, and all wetted parts are constructed from Alloy C-276 to prevent degradation. K type thermocouple located immediately behind the trough within the body of the cell. MCT detector used for analysis.

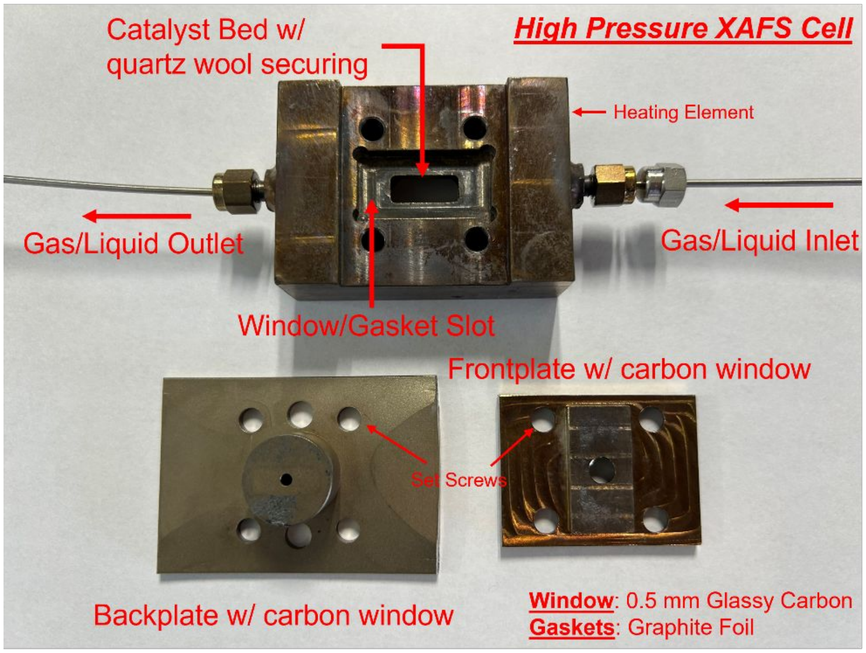

Fig. S29. Detailed schematic of high pressure in situ XAFS cell. Pressure is applied via two backpressure regulators for a working total of 34 bar, catalyst secured in bed with quartz plugs. Measurement Conditions: 34 bar pressure total, either pure DI H_2O flow or 0.1 M H_2O_2 (aq) flow saturated with 20 bar of methane, temperature varied between 25 – 90 °C, 0.1 mL/min total flow via HPLC pump, PIPs fluorescence detector, beamline 8-ID flux.
